# Synthesis and Reactivity of Dipalladated Derivatives of Terephthalaldehyde

**DOI:** 10.1021/acs.organomet.4c00231

**Published:** 2024-07-24

**Authors:** María-José Fernández-Rodríguez, Peter G. Jones, José Vicente, Eloísa Martínez-Viviente

**Affiliations:** † Grupo de Química Organometálica, Departamento de Química Inorgánica, Facultad de Química, Universidad de Murcia, Murcia E-30071, Spain; ‡ Institut für Anorganische und Analytische Chemie, Technische Universität Braunschweig, Hagenring 30, Braunschweig 38106, Germany

## Abstract

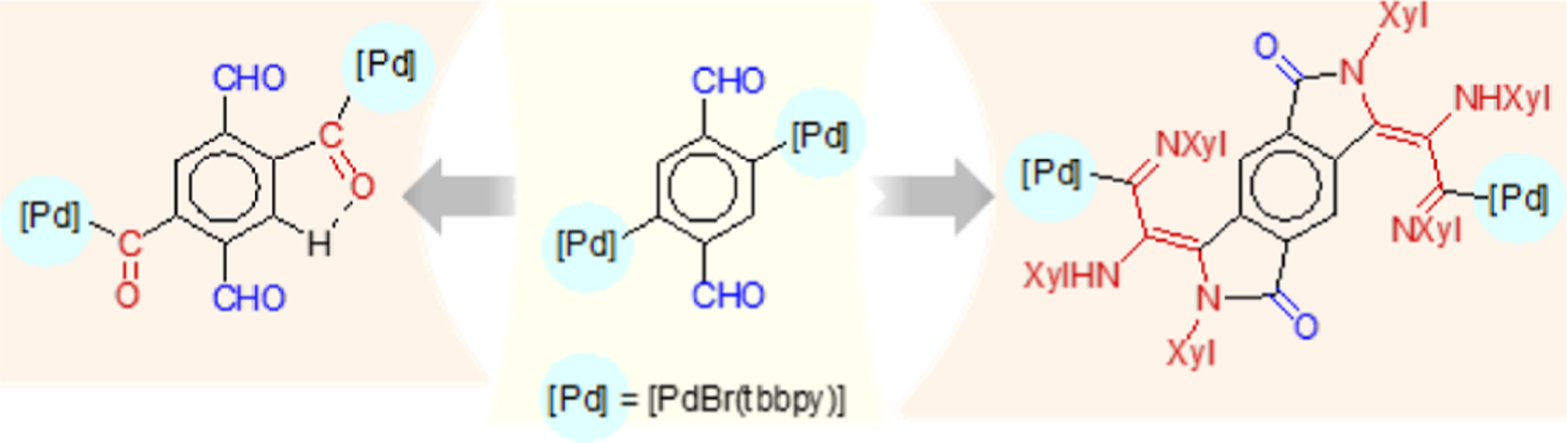

The polynuclear complex [{μ-*C*1,*C*4,*N*,*N*″-C_6_H_2_{C(H)=N(^n^Bu)}_2_-2,5}{Pd(μ-OAc)}]_2_ (**I**) reacts with tbbpy (4,4′-di-*tert*-butyl-2,2′-bipyridine) and TlOTf to form the dinuclear complex [{μ-*C*1,*C*4,*N*,*N*″-C_6_H_2_{C(H)=N(^n^Bu)}_2_-2,5}{Pd(tbbpy)}_2_] (**1**). The hydrolysis of **I** with acetic acid in a 5:1 acetone/water mixture, in the presence of two equivalents of tbbpy and excess NaX (X = Br, I), yields the dipalladated terephthalaldehyde complexes [C_6_H_2_{PdX(tbbpy)}_2_-1,4-(CHO)_2_-2,5] [X = Br (**2a**), X = I (**2b**)], which are the first fully characterized complexes of this type. The reaction of **2a,b** with CO results in the insertion of CO into both aryl–Pd bonds, forming [C_6_H_2_{C(O){PdX(tbbpy)}}_2_-1,4-(CHO)_2_-2,5] [X = Br (**3a**), X = I (**3b**)], which are the first examples of complexes with CO inserted into two separate aryl–metal bonds involving the same ligand. The bromo complex **2a** reacts with excess XylNC in acetone, causing the precipitation of the dinuclear complex 2,3,6,7-tetrahydrobenzo[1,2-*c*:4,5-*c*′]dipyrrole-1,5-dione-2,6-dixylyl-3,7-bis{=C(NHXyl)-C(=NXyl)-[PdBr(CNXyl)_2_]} (**4**), which is the result of the insertion of three molecules of the isocyanide into each aryl–Pd bond and the nucleophilic attack of one of them at each formyl group. When complex **4** reacts with TlOTf and residual water in 1,2-dichloroethane at 70 °C, depalladation occurs, and the organic compound 2,3,6,7-tetrahydrobenzo[1,2-*c*:4,5-*c*′]dipyrrole-1,5-dione-2,6-dixylyl-3,7-bis{=C(NHXyl)–C(O)NHXyl} (**5**) can be isolated. The crystal structures of **1**·4CHCl_3_, **4**·2CH_2_Cl_2_·3hexane, and **5**·2CDCl_3_ have been determined by X-ray crystallography.

## Introduction

**Chart 1:**
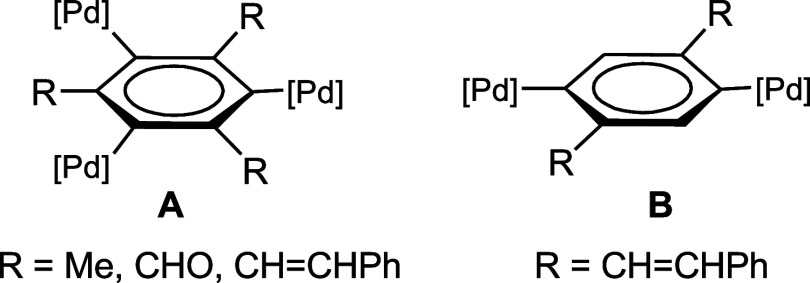


Arylpalladium complexes are crucial intermediates in a wide range of palladium-catalyzed carbon–carbon and carbon-heteroatom bond-forming reactions, which have facilitated the efficient and selective construction of complex organic molecules.^1−18^ Consequently, the synthesis, characterization, and reactivity of these complexes remain a very active field of research.^19−41^ Organic substituents *ortho* to the Pd atom may be involved in this chemistry, yielding novel structures and potentially useful organic compounds.^21−30,34,35^ We have been interested in exploring the extension of this chemistry to di-^42,43^ and tripalladated^44−47^ benzene derivatives *ortho*-substituted at each Pd(II) center. Thus, our group has reported the synthesis and reactivity of mono-, di-, and tripalladated derivatives of mesitylene,^44,46^ benzenetricarboxaldehyde,^45^ and tris(styryl)benzene^47^ (Chart 1A), as well as dipalladated derivatives of 2,5-distyrylbenzene^42,43^ (Chart 1B).

Whereas the trinuclear complexes depicted in Chart 1 are fairly rare,^44−47^ reports of dinuclear *ortho*-substituted arylpalladium complexes are less scarce, although they refer, with some exceptions,^42,43,48^ to dipalladacycles with N-donor groups.^49−57^ We report now the first fully characterized dipalladated derivatives of terephthalaldehyde (R=CHO in Chart 1B) and a preliminary investigation of their reactivity toward unsaturated molecules (CO and XylNC). These reactions have resulted in the first complexes resulting from the double insertion of CO into two separate aryl–metal bonds of the same aryl ring, as well as the synthesis of a novel dinuclear Pd(II) complex derived from a multiple XylNC insertion, which can be depalladated to yield an interesting polycyclic organic compound containing a benzodipyrrole-1,5-dione core.

## Results and Discussion

### Synthesis

**Scheme 1:**
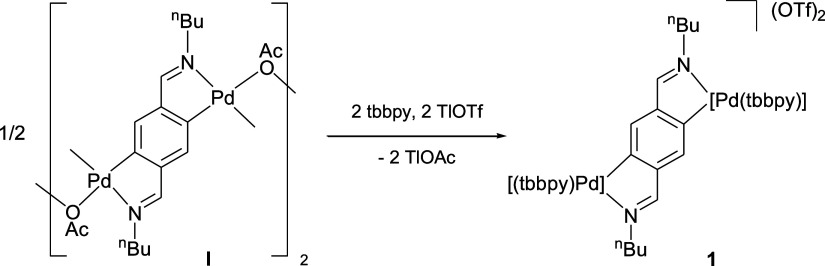
Complex **I** Reacts with tbbpy and TlOTf to Form the Dipalladated Complex **1**

The polynuclear complex [{μ-*C*1,*C*4,*N*,*N*″-C_6_H_2_{C(H)=N(^n^Bu)}_2_-2,5}{Pd(μ-OAc)}]_2_ (**I**, Scheme 1) had been previously prepared in our research group by palladation of the diimine C_6_H_4_(CH=N^n^Bu)_2_-1,4 with [Pd(OAc)_2_].^58^ Complex **I** is soluble in common solvents, in contrast to a similar complex with Tol instead of ^n^Bu, also prepared in our research group.^51^ The reaction of **I** with tbbpy (4,4′-di-*tert*-butyl-2,2′-bipyridine) and TlOTf results in the dinuclear complex [{μ-*C*1,*C*4,*N*,*N*″-C_6_H_2_{C(H)=N(^n^Bu)}_2_-2,5}{Pd(tbbpy)}_2_] (**1**, Scheme 1). Complexes **I** and **1** are of interest because they contain an aryl ligand capable of binding two different metal centers simultaneously in a tetradentate fashion, resulting in two independent palladacycles on the same aryl ring. Such complexes are still relatively rare in the literature, although some examples can be found involving mainly N-donor groups.^49−57^ The examples most closely related to our work are the dipalladated Schiff bases reported by Vila and co-workers, prepared by palladation or oxidative addition reactions, followed by ligand exchange.^55,56^ These dinuclear square-planar palladium(II) complexes with two blocked *cis*-coordination sites can be very useful as building blocks in supramolecular chemistry.^55,59^

**Scheme 2:**
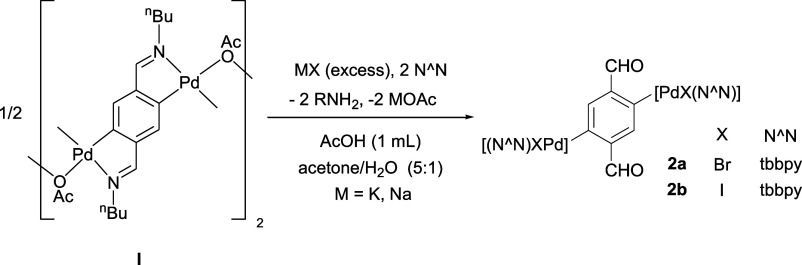
Hydrolysis of Complex **I** to Form the Dipalladated Complexes **2a,b**

The hydrolysis of **I** with acetic acid in a 5:1 acetone/water mixture, in the presence of two equivalents of tbbpy and excess NaX (X = Br, I), yields the dipalladated terephthalaldehyde complexes [C_6_H_2_{PdX(tbbpy)}_2_-1,4-(CHO)_2_-2,5] [X = Br (**2a**), X = I (**2b**), Scheme 2]. These are the first such dinuclear Pd complexes to be fully characterized, as in a previous attempt by our research group,^51^ starting from a complex similar to **I** with Tol instead of ^n^Bu, and using bpy instead of tbbpy as the ligand, the formation of a similar complex [C_6_H_2_{PdBr(bpy)}_2_-1,4-(CHO)_2_-2,5] was proposed, but it was too insoluble to be purified and characterized. Similar reactions with tmeda instead of tbbpy have resulted in mixtures of compounds that could not be separated.

**Scheme 3:**
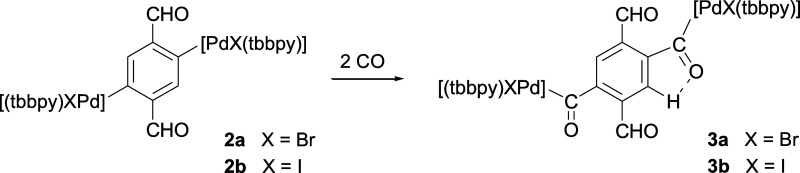
CO Insertion in **2a,b** to Form **3a,b**

The reaction of **2a,b** with CO results in the complexes [C_6_H_2_{C(O){PdX(tbbpy)}}_2_-1,4-(CHO)_2_-2,5] [X = Br (**3a**), X = I (**3b**), Scheme 3], resulting from the insertion of CO into both aryl–Pd bonds of **2a,b** (Scheme 3). These are the first reported complexes resulting from the insertion of CO into two separate aryl–metal bonds involving the same aryl ligand. The infrared (IR) and nuclear magnetic resonance (NMR) spectra of **3a,b** confirm the insertion of CO (see below). Additionally, the NMR spectra suggest that one of the inserted CO groups forms a hydrogen bond with the adjacent aromatic hydrogen, while the other inserted CO does not behave similarly. The syntheses of **3a,b** are best carried out in distilled THF (heating to 60 °C for several hours, see Experimental Section). In CH_2_Cl_2_ or 1,2-dichloroethane, the reactions are much less clean.

**Scheme 4:**
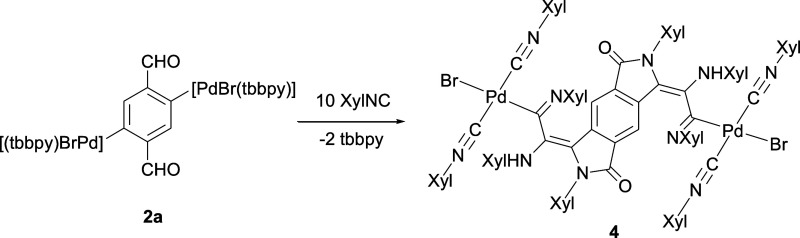
Reaction of **2a** with XylNC to Form the Dinuclear Complex **4**

**Scheme 5:**
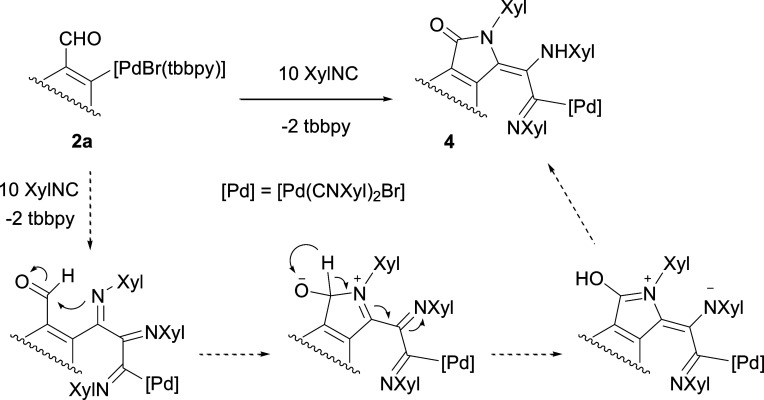
Proposed Mechanism for the Reaction of **2a** with XylNC to Form **4**

When bromo complex **2a** reacts with a 2-fold excess of XylNC in acetone, singular dinuclear complex 2,3,6,7-tetrahydrobenzo[1,2-*c*:4,5-*c*′]dipyrrole-1,5-dione-2,6-dixylyl-3,7-bis{=C(NHXyl)-C(=NXyl)-[PdBr(CNXyl)_2_]} (**4**) precipitates as a red solid (Scheme 4). Complex **4** is the result of the insertion of three molecules of the isocyanide into each aryl–Pd bond and the nucleophilic attack of one of them at each formyl group, followed by an intramolecular proton migration (Scheme 5). The tbbpy ligands on the Pd atoms are displaced by two other XylNC molecules, which, as usual, adopt a *trans* disposition.^23,24,27,43^ It would seem that the insolubility of **4** plays an important role in its formation as similar reactions with iodo complex **2b**, or with ^t^BuNC instead of XylNC, result in mixtures of compounds. Complex **4** decomposes slowly in solution to give [PdBr_2_(XylNC)_4_], which is easily identified by its ^1^H NMR resonance at 2.52 ppm.^23^

**Scheme 6:**
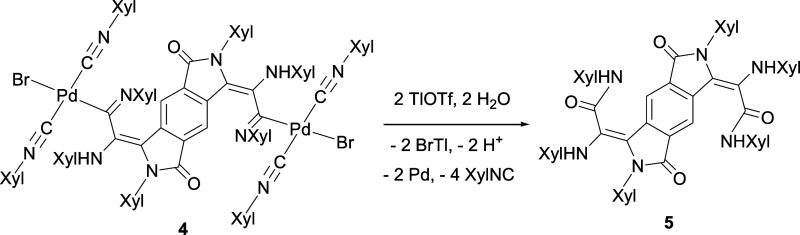
Depalladation of **4** to Form the Organic Compound **5**

When complex **4** reacts with TlOTf in 1,2-dichloroethane at 70 °C, depalladation occurs, forming the new organic compound 2,3,6,7-tetrahydrobenzo[1,2-*c*:4,5-*c*′]dipyrrole-1,5-dione-2,6-dixylyl-3,7-bis{=C(NHXyl)–C(O)NHXyl} (**5**, Scheme 6). In this reaction, the precipitation of TlBr promotes the substitution of both [PdBr(XylNC)_2_] moieties by hydroxyl groups from residual water molecules, followed by a tautomeric equilibrium, resulting in a benzodipyrrole-1,5-dione core with two alkylidene substituents at positions 2 and 6. There is no other synthetic route described for the synthesis of a compound such as **5**, and even for related, although more simple, benzodipyrrolediones, we have only found two precedents in the literature, none of them involving Pd: a report on cobalt-catalyzed carbonylation of Schiff bases as a step for the synthesis of benzenetetracarboxylic acids^60^ and the organic synthesis of bis(hydroxy-isoindolinones) as building blocks for supramolecular assembly.^61^

### Structure of Complexes

Complex **1** shows the expected IR bands for the imine C=N bond (1614 cm^–1^) and the S=O bond of the OTf anion (1030, 1280 cm^–1^). In solution, **1** shows fluxional behavior, resulting in broad NMR resonances for the tbbpy ligand. This dynamic process might be promoted by the coordination of the OTf anion to the Pd atom, leading to a five-coordinated intermediate where the two halves of the tbbpy ligand would exchange by dissociation of one of the N atoms, rotation around the remaining Pd–N bond, and recoordination to Pd. We have described a similar process in anionic dinuclear indacenediide palladium complexes, also featuring a tbbpy ligand and an OTf anion.^43^

Complexes **2a,b** show the expected IR bands for the formyl C=O bonds at 1672 and 1662 cm^–1^, respectively, while for **3a,b**, we observe a broad band at 1682 cm^–1^ (**3a**) or two bands at 1662 and 1678 cm^–1^ (**3b**), confirming the presence of additional C=O groups resulting from the insertion of CO into both aryl–Pd bonds. 1D and 2D NMR spectra of **2a,b** and **3a,b** have allowed full assignment of their ^1^H and ^13^C resonances (see Experimental Section). **2a,b** complexes show a single set of ^1^H and ^13^C resonances for the two halves of the molecule, which are made equivalent by an inversion center in solution. For **3a,b**, interestingly, there is no such symmetry, and two well-separated ^1^H and ^13^C resonances for the two CH groups of the aryl ring are observed, while all of the other resonances of the two halves of the molecule coincide (see Table 1). This inequivalence of the two CH groups in **3a,b** could be explained by the formation of a hydrogen bond between one of the inserted CO groups and the adjacent aryl proton, while the same would not happen for the other CO group, as a result of steric or electronic reasons. The aryl hydrogen involved in the hydrogen bond (H3″) in **3a,b** would be shifted to higher frequencies (δ 8.48 ppm) with respect to the other aryl hydrogen, which would resonate at a similar frequency (δ 8.1 ppm) as in **2a,b** (data in blue in Table 1). The ^13^C resonances of CH3 and CH3″ would also be affected, while for the other carbons in **3a,b**, the difference in chemical shift would be so small that it would not be observable.^a^ As also shown in Table 1, **2a,b** and **3a,b** show the expected ^1^H and ^13^C NMR resonances for the CHO groups. However, the ^13^C resonances of the inserted CO groups were not observed in **3a,b**, possibly because of the long relaxation times. Nonetheless, the insertion of the CO molecules is confirmed by the elemental analyses and also by the IR spectra (as mentioned above).

**Table 1:**
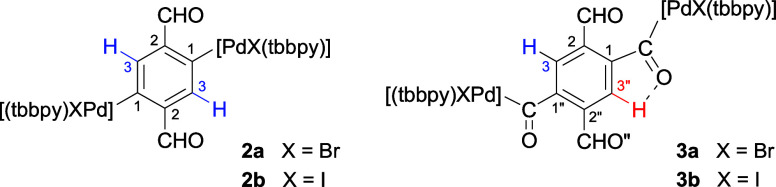
Selected ^1^H and ^13^C NMR Data (ppm) for **2a,b** and **3a,b**

	**2a**		**2b**			**3a**		**3b**	
	^**13**^**C**	^**1**^**H**	^**13**^**C**	^**1**^**H**		^**13**^**C**	^**1**^**H**	^**13**^**C**	^**1**^**H**
**C1**	152.6		149.2		**C1**	168.3		165.7	
**C2**	143.9		143.6		**C2**	138.7		138.9	
**CH**	135.8	8.12	136.6	8.11	**CH3″**	144.6	8.48	146.5	8.48
					**CH3**	128.5	8.14	128.3	8.14
**CHO**	197.4	11.10	197.8	11.03	**CHO**	196.3	11.01	196.8	10.95

The IR spectrum of complex **4** shows the expected bands for the N–H bond (3376 cm^–1^), the C≡N bonds of the coordinated isocyanides (2182 cm^–1^), the carbonyl C=O bonds (1682 cm^–1^), and the C=N bond of the inserted isocyanide (1614 cm^–1^). For the organic compound **5**, we observe an N–H band at 3369 cm^–1^ and a broad C=O band at 1674 cm^–1^. The ^1^H and ^13^C NMR resonances of both **4** and **5** were also assigned with the help of 1D and 2D spectra. Both complexes feature an inversion center in solution (as in the solid state) so that the halves of the molecules are equivalent, and only one set of ^1^H and ^13^C NMR resonances is observed. The NMR data also indicate that there is free rotation around all the N–Xyl bonds, making both Me groups on each ring equivalent. For complex **4**, the two XylNC ligands on each Pd are also equivalent, confirming the *trans* geometry proposed for this complex.

^1^ Indeed, the APT spectrum of **2b**, when processed without window function, shows a significant broadening of the ^13^C resonances of the CHO/CHO″, C1/C1″, and C2/C2″ carbons but not of those of the tbbpy ligand.

**Figure 1:**
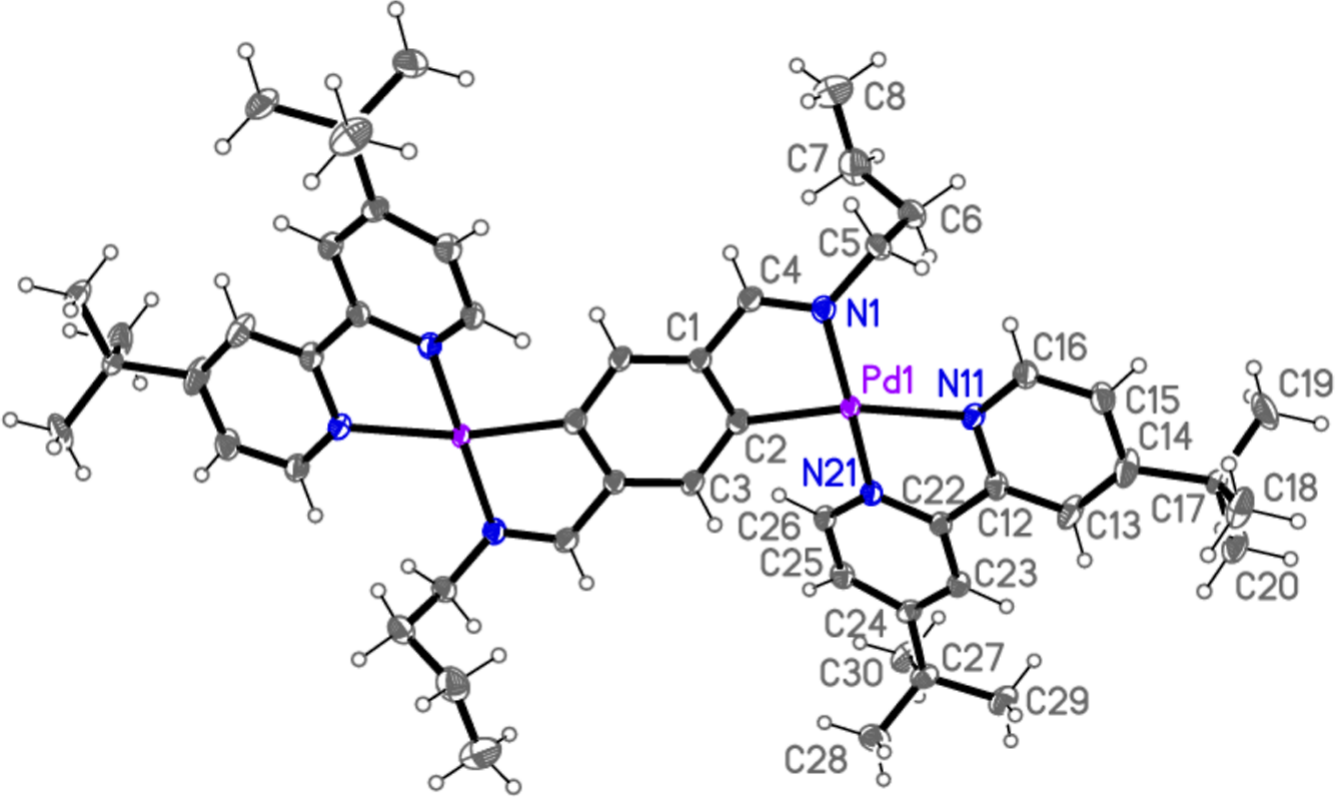
Thermal ellipsoid plot (50% probability level) of **1**·4CHCl_3_. Only the cation is shown, and only the asymmetric unit is numbered. Selected bond lengths (Å) and angles (deg): Pd–C(2) = 2.002(5), Pd–N(1) = 2.058(5), Pd–N(21) = 2.039(5), Pd–N(11) = 2.160(4), C(1)–C(2) = 1.428(7), C(1)–C(4) = 1.428(7), N(1)–C(4) = 1.291(7), C(2)–Pd–N(1) = 80.3(2), C(2)–Pd–N(21) = 98.5(2), N(21)–Pd–N(11) = 78.14(17), N(1)–Pd–N(11) = 104.84(18), N(21)–Pd–N(1) = 172.26(19), C(2)–Pd–N(11) = 165.78(19), C(4)–C(1)–C(2) = 114.1(5), C(1)–C(2)–Pd = 113.1(4), N(1)–C(4)–C(1) = 117.8(5), C(4)–N(1)–Pd = 114.6(4).

**Figure 2:**
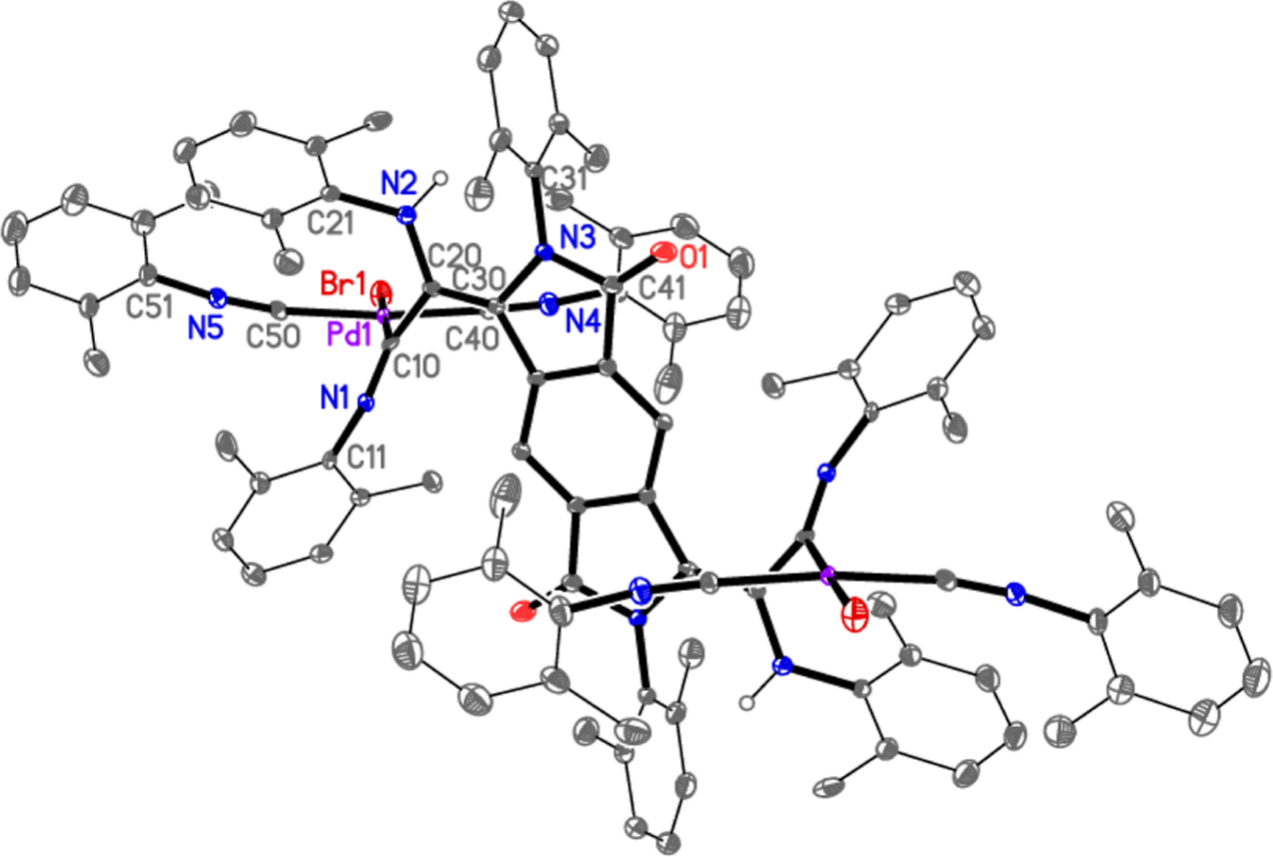
Thermal ellipsoid plot (30% probability level) of **4**·2CH_2_Cl_2_·3hexane. The solvent is omitted as are all hydrogen atoms except that at N2, and only the asymmetric unit is numbered. Selected bond lengths (Å) and angles (deg): Pd–C(40) = 1.969(8), Pd–C(50) = 1.970(8), Pd–C(10) = 2.041(7), Pd–Br = 2.5338(10), N(1)–C(10) = 1.262(9), N(2)–C(20) = 1.389(9), N(3)–C(30) = 1.428(9), C(10)–C(20) = 1.476(10), C(20)–C(30) = 1.364(10), O–C(4) = 1.215(9), N(3)–C(4) = 1.387(10), C(40)–Pd–C(10) = 89.8(3), C(50)–Pd–C(10) = 92.4(3), C(40)–Pd–Br = 88.5(2), C(50)–Pd–Br = 89.8(2), C(40)–Pd–C(50) = 174.0(3), C(10)–Pd–Br = 174.9(2), C(10)–N(1)–C(11) = 126.8(6), C(20)–N(2)–C(21) = 125.1(6), C(4)–N(3)–C(30) = 112.7(6), C(4)–N(3)–C(31) = 118.3(6), C(30)–N(3)–C(31) = 129.0(6).

**Figure 3:**
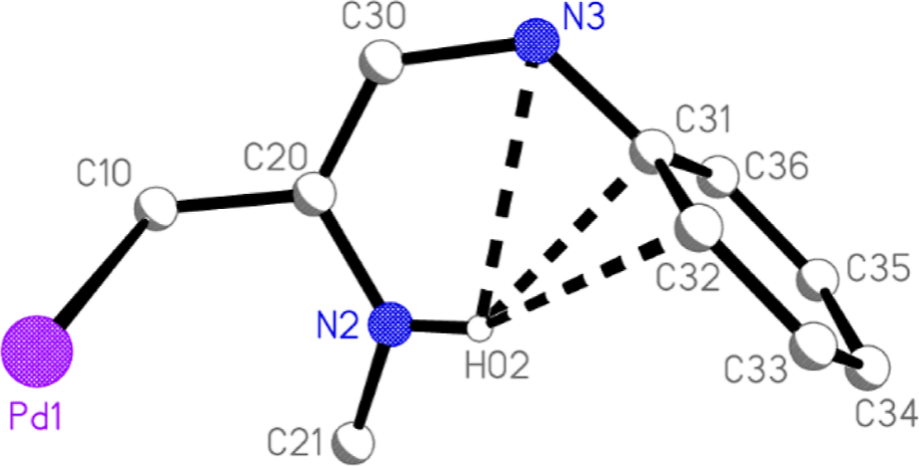
Intramolecular C–HΛπ hydrogen bonding system in **4**·2CH_2_Cl_2_·3hexane. Thick dashed lines represent the three shortest contacts.

**Figure 4:**
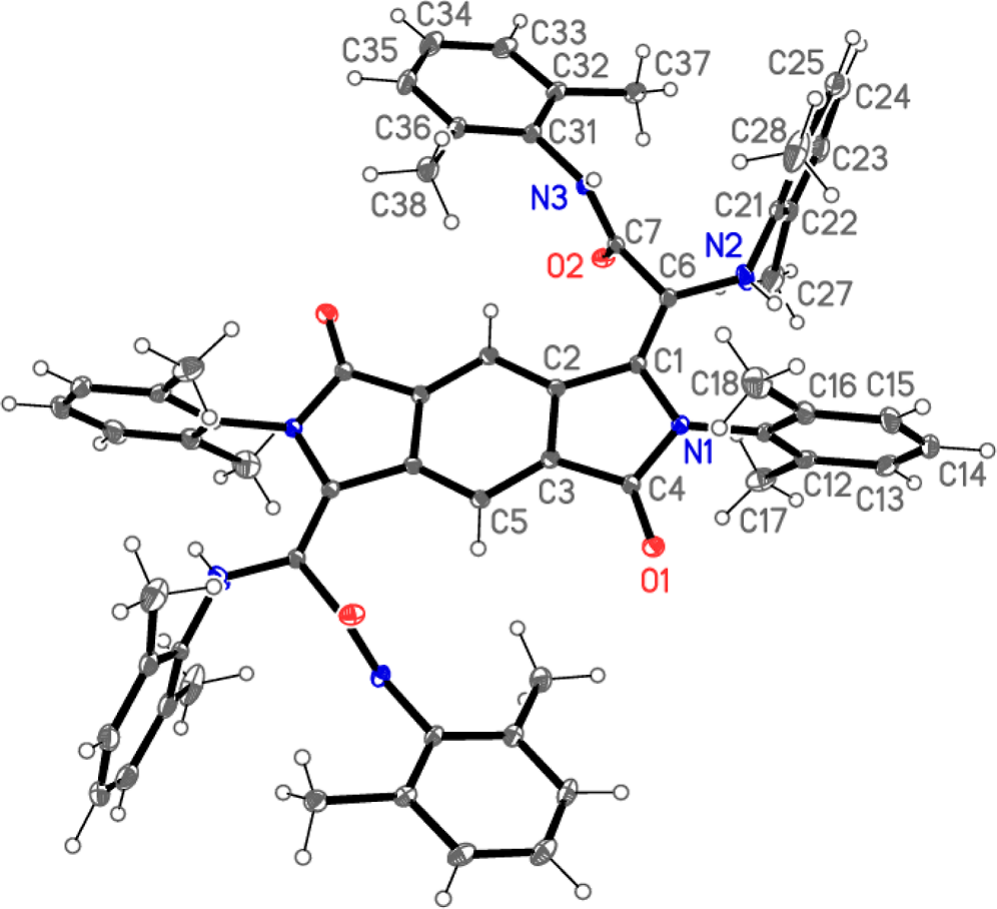
Thermal ellipsoid plot (30% probability level) of **5**·2CDCl_3_. The solvent is omitted, and only the asymmetric unit is numbered. Selected bond lengths (Å) and angles (deg): C(1)–C(6) = 1.357(2), C(1)–N(1) = 1.427(2), C(1)–C(2) = 1.462(2), C(4)–O(1) = 1.225(2), C(4)–N(1) = 1.380(2), C(4)–C(3) = 1.467(2), C(6)–N(2) = 1.372(2), C(6)–C(7) = 1.522(2), C(7)–O(2) = 1.220(2), C(7)–N(3) = 1.354(2), C(6)–C(1)–N(1) = 125.80(12), C(6)–C(1)–C(2) = 128.67(12), N(1)–C(1)–C(2) = 105.35(11), O(1)–C(4)–N(1) = 125.13(13), O(1)–C(4)–C(3) = 128.96(13), N(1)–C(4)–C(3) = 105.88(12), C(1)–C(6)–N(2) = 124.75(13), C(1)–C(6)–C(7) = 118.67(12), N(2)–C(6)–C(7) = 116.50(12), O(2)–C(7)–N(3) = 123.66(13), O(2)–C(7)–C(6) = 120.29(13), N(3)–C(7)–C(6) = 116.04(12), C(4)–N(1)–C(1) = 112.17(11), C(4)–N(1)–C(11) = 120.43(12), C(1)–N(1)–C(11) = 127.30(12), C(6)–N(2)–C(21) = 125.75(13), C(7)–N(3)–C(31) = 122.54(12).

**Figure 5:**
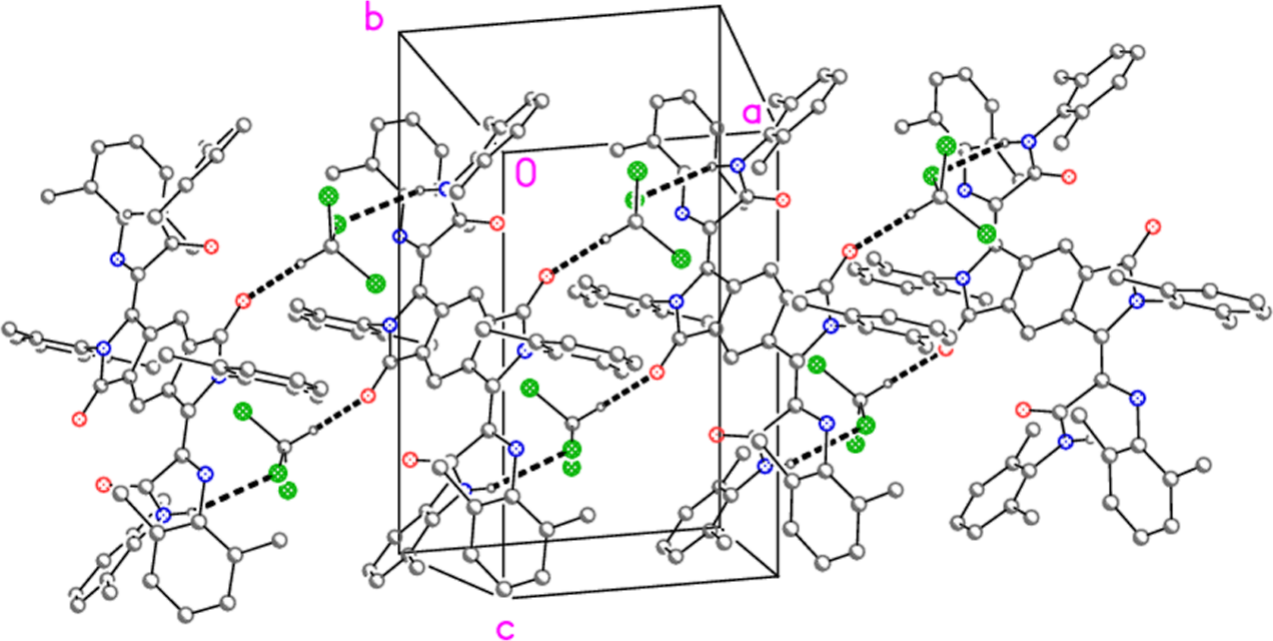
Hydrogen bonding system in **5**·2CDCl_3_. Thick dashed lines represent hydrogen bonds. Hydrogen atoms not involved in hydrogen bonding have been omitted for clarity. Only the major component (83%) of the disordered solvent is shown.

The crystal structures of **1**·4CHCl_3_ (Figure 1), **4**·2CH_2_Cl_2_·3hexane (Figures 2 and 3), and **5**·2CDCl_3_ (Figures 4 and 5) have been determined by X-ray diffraction studies (Table 1 in Supporting Information). The structure of **1**·4CHCl_3_ (Figure 1) shows the crystallographic inversion symmetry of **1** and confirms the doubly chelating nature of the diimine ligand. The chelate ring is to a good approximation planar, with a mean deviation of 0.014 Å. The coordination of the iminic nitrogen to Pd leads to a slight lengthening of the C=N bond [1.291(7) Å] with respect to the mean value in imines (1.279 Å).^62^ The coordination around the Pd atoms is square planar but is markedly distorted to avoid a close contact between H3 and H26 (for which the observed distance is 2.12 Å); the atoms Pd, N11, N21, and N1 are coplanar (mean deviation 0.04 Å), but C2 lies 0.64 Å outside the plane thus defined. The Pd–C bond distance is 2.002(5) Å, similar to the values reported by some of us for other aryl palladium complexes with an aryl ligand *trans* to bpy or tbbpy (ca. 1.97–2.00 Å).^20,27,46,63^ The three Pd–N bond distances follow the expected order of the *trans* influence: Pd–N *trans* to aryl [2.160(4) Å] > Pd–N *trans* to N [2.039(5) and 2.058(5) Å].

The structure of complex **4**·2CH_2_Cl_2_·3hexane is also confirmed by its X-ray diffraction study (Figure 2) and, similarly to **1**·4CHCl_3_, shows the crystallographic inversion symmetry of **4**. Unfortunately, the large amount of included solvent (with high *U* values for the hexanes) leads to data with a relatively low resolution. The Pd atoms in **4**·2CH_2_Cl_2_·3hexane show square planar coordination to a reasonable approximation with a mean deviation from the best plane through Pd and the four donor atoms of 0.07 Å. The Pd–C(10) bond distance of the iminoacyl ligand is 2.041(7) Å, significantly longer than that in the aryl palladium complex **1**·4CHCl_3_ [2.002(5) Å, see above]. Pd–C bond distances in iminoacyl ligands are known to vary considerably, depending on the influence of the *trans* ligand.^23,64−66^ For the isocyanide ligands, the Pd–C(40) and Pd–C(50) bond distances are 1.969(8) and 1.970(8) Å, similar to the values found in other Pd complexes with two mutually *trans* XylNC ligands (ca. 1.96–1.99 Å in those reported by some of us).^23,64−67^ Finally, the Pd–Br bond distance of 2.5338(10) Å is similar to those observed in other complexes where the Br ligand is *trans* to an iminoacyl ligand.^23,26,68^

At first sight, the NH group does not seem to be involved in hydrogen bonding. However, closer inspection reveals a close intramolecular approach of the NH hydrogen atom to the atoms N3, C31, C32, and C36 of a neighboring ring, with distances of 2.65(7), 2.24(5), 2.49(4), and 2.85(5) Å, respectively (Figure 3). This may reasonably be regarded as a C–HΛπ interaction. The dichloromethane molecule, which is ordered, makes a very short contact (a “weak” hydrogen bond) with C–HΛO1 of only 2.20 Å.

The structure of organic compound **5**·2CDCl_3_ has also been confirmed by its X-ray diffraction study (Figure 4). Again, main molecule **5** shows crystallographic inversion symmetry. The data for both **4**·2CH_2_Cl_2_·3hexane and **5**·2CDCl_3_ suggest a delocalization of π electron density along the N–C(4)=O bonds within the five-membered ring based on the short N–C(4) bond distance (ca. 1.38 Å), compared with the adjacent N(3)–C(30) [1.428(9) Å, **4**·2CH_2_Cl_2_·3hexane] or N(1)–C(1) [1.427(2) Å **5**·2CDCl_3_] bonds within the same ring. The three angles around the N atom within the five-membered ring also support this suggestion as their values for both compounds are ca. 112°, 118–120°, and 127–129° for the three angles, respectively.

We also find a delocalization of π electron density along the N(2)–C=C bonds based on the short N(2)–C(20) [1.389(9) Å, **4**·2CH_2_Cl_2_·3hexane] or N(2)–C(6) [1.372(2) Å, **5**·2CDCl_3_] bond lengths, which are intermediate between the values for the double bond N(1)=C(10) in **4**·2CH_2_Cl_2_·3hexane [1.262(9) Å] and the single N–Xyl bonds (ca. 1.42–1.43 Å in both compounds). The wide C–N(2)–C(21) angles (ca. 125°) and the short C=C bonds [C(30)–C(20) or C(1)–C(6), both ca. 1.36 Å] compared with the adjacent C(30)–C(2) [1.471(10) Å, **4**·2CH_2_Cl_2_·3hexane] or C(1)–C(2) [1.462(2) Å, **5**·2CDCl_3_] bonds also support this suggestion.

The delocalization of π electron density is also reflected in the almost planar arrangement of O(1), the heterocyclic core, the aliphatic chain, and N(2). Thus, in **4**·2CH_2_Cl_2_·3hexane, the atoms C(1)–C(2)–C(3)–C(4)–O–N(3)-C(31)–C(30)–C(20)–C(10)–N(2)–C(21) are almost coplanar (mean deviation 0.07 Å) as are (to a lower degree) the atoms C(5)–C(3)–C(2)–C(4)–O(1)–N(1)–C(31)–C(1)–C(6)–C(7)–N(2)–C(21) in **5**·2CDCl_3_ (here the mean deviation is larger, 0.17 Å).

In organic compound **5**·2CDCl_3_, we also find a delocalization of electron density over the bonds N(3)–C(7)–O(2), as shown by the coplanarity of the group of atoms C(31)–N(3)–C(7)–O(2)–C(6), with a mean deviation of 0.02 Å. The angle between this plane and the major plane described in the previous paragraph is 89.5°. The N(3)–C(7) bond length, 1.354(2) Å, is even shorter than the N(1)–C(4) [1.380(2) Å] and N(2)–C(6) bond lengths [1.372(2) Å]. The carbonyl C=O bond lengths [C(7)–O(2), 1.220(2) Å; C(4)–O(1), 1.225(2) Å] are as expected for C_sp2_=O amides.^62^

Similarly to **4**·2CH_2_Cl_2_·3hexane, the hydrogen atom of one NH group makes short intramolecular contacts to a neighboring ring, with H02ΛC11 2.32(2), H02ΛC12 2.75(2), H02ΛC16 2.72(2), and H02ΛN1 2.69(2) Å. The other NH group is hydrogen-bonded to a solvent chlorine atom [H03ΛCl2 2.94(2) Å, symmetry operator 1–*x*, 1–*y*, and 1–*z*], and the solvent CD group is hydrogen-bonded to O1, with a DΛO distance of only 2.05 Å. The hydrogen bonds combine to form broad ribbons of residues parallel to the *a* axis (Figure 5).

## Conclusions

We have described the first fully characterized dipalladated derivatives of terephthalaldehyde and also their reactivity toward CO and XylNC, which has resulted in the first reported complexes featuring CO insertion into two separate aryl–metal bonds on the same ligand, as well as the double 3-fold insertion of XylNC, leading to the synthesis of a singular dipalladated benzodipyrrole-1,5-dione derivative, which can be depalladated to yield the free polycyclic organic ligand. This chemistry showcases the great potential of polypalladated arenes for the synthesis of complex organic compounds.

## Experimental Section

NMR spectra (^1^H and ^13^C) were recorded on 400 and 600 MHz Bruker AVANCE spectrometers at room temperature. Chemical shifts are given in parts per million (δ) relative to TMS (^1^H, ^13^C). IR spectra were recorded on a PerkinElmer 16F-PC-FT spectrometer with Nujol mulls between polyethylene sheets. Melting points were determined on a Reichert apparatus and are uncorrected. Elemental analyses were carried out with a Carlo Erba 1106 microanalyzer. Experiments under a N_2_ atmosphere were conducted using standard Schlenk techniques. THF, CH_2_Cl_2_, and Et_2_O were degassed and dried by using a Pure Solv MD-5 solvent purification system from Innovative Technology Inc. [{μ-*C*1,*C*4,*N*,*N*″-C_6_H_2_{C(H)=N(^n^Bu)}_2_-2,5}{Pd(μ-OAc)}]_2_ (**I**) was prepared from the diimine C_6_H_4_{C(H)=N(^n^Bu)}_2_-1,4 (generated in situ from terephthalaldehyde and ^n^BuNH_2_) by palladation with [Pd(OAc)_2_], as previously described^58^ (see Supporting Information). TlOTf was prepared by the reaction of Tl_2_CO_3_ and triflic acid (1:2) in water and recrystallized from acetone/Et_2_O. [Pd(dba)_2_] was prepared according to a literature procedure.^69,70^ All other reagents were obtained from commercial sources and used as received.

### Synthesis of [{μ-*C*1,*C*4,*N*,*N*″-C_6_H_2_{C(H)=N(^n^Bu)}_2_-2,5}{Pd(tbbpy)}_2_](OTf)_2_ (**1**)

TlOTf (123 mg, 0.35 mmol) and tbbpy (93 mg, 0.35 mmol) were added to a solution of **I** (100 mg, 0.087 mmol) in CH_2_Cl_2_. The mixture was stirred for 16 h at room temperature (the color changed from reddish to yellow). Then, it was filtered through Celite, and the resulting yellow solution was evaporated to dryness. Et_2_O (20 mL) was added to precipitate a solid, which was filtered off, washed with Et_2_O (3 × 5 mL), and dried in vacuo to give **1** as a yellow solid. Yield: 206 mg (92%). Mp: 204 °C. Λ_M_ (acetone): 143 Ω^–1^ cm^2^ mol^–1^. IR (cm^–1^): ν(C=N) 1614, ν(S=O) 1030, 1280. ^**1**^**H NMR (400 MHz, CDCl**_**3**_**):** δ 9.08 (br s, 2H, tbbpy), 8.74 (s, 2H, HC=N), 8.57 (br s, 2H, tbbpy), 8.20–7.95 (br m, 6H, tbbpy), 7.69 (br s, 2H, tbbpy), 7.49 (s, 2H, H3 aryl), 3.92 (q, ^3^*J*_HH_ = 7, 4H, CH_2_^n^Bu), 1.83 (quint, ^3^*J*_HH_ = 7, 4H, CH_2_^n^Bu), 1.52 (m, 4H, CH_2_^n^Bu), 1.49 (s, 36H, ^*t*^Bu tbbpy), 0.96 (t, ^3^*J*_HH_ = 7, 6H, CH_3_^n^Bu). Anal. Calcd for C_54_H_70_F_6_N_6_O_6_Pd_2_S_2_: C, 50.27; H, 5.47, N, 6.51; S, 4.97. Found: C, 50.03; H, 5.58; N, 6.17; S, 4.73. Single crystals of **1·**4CHCl_3_ were grown by liquid diffusion of Et_2_O into a solution of **1** in CHCl_3_.

### Synthesis of [C_6_H_2_{PdBr(tbbpy)}_2_-1,4-(CHO)_2_-2,5] (**2a**)

Complex **I** (500 mg, 0.44 mmol), NaBr (897 mg, 8.72 mmol), and AcOH (1 mL) were added to a solution of tbbpy (467 mg, 1.74 mmol) in a 72 mL mixture of acetone and water (5:1), and the resulting suspension was refluxed for 6 h. A solid formed, which was filtered off and then washed with water (3 × 10 mL) and a small amount of acetone (2 mL). The solid was then redissolved in CH_2_Cl_2_ (20 mL), stirred with MgSO_4_ for 30 min, and then filtered through additional MgSO_4_, yielding a yellow solution, which was evaporated to dryness. Et_2_O (20 mL) was added to precipitate a solid, which was filtered off, thoroughly washed with Et_2_O (3 × 5 mL), and dried in vacuo to give **2a** as a yellow solid. Yield: 852 mg (94%). Mp: 262 °C. IR (cm^–1^): ν(C=O): 1672.^**1**^**H NMR (400 MHz, CDCl**_**3**_**):** δ 11.10 (s, 2H, CHO), 9.31 (d, ^3^*J*_HH_ = 6, 2H, H16′ tbbpy), 8.12 (s, 2H, H3 aryl), 8.00 (d, ^4^*J*_HH_ = 2, 2H, H13′ tbbpy), 7.98 (d, ^4^*J*_HH_ = 2, 2H, H13 tbbpy), 7.57 (dd, ^3^*J*_HH_ = 6, ^4^*J*_HH_ = 2, 2H, H15′ tbbpy), 7.55 (d, ^3^*J*_HH_ = 6, 2H, H16 tbbpy), 7.33 (dd, ^3^*J*_HH_ = 6, ^4^*J*_HH_ = 2, 2H, H15 tbbpy), 1.45 (s, 18H, ^*t*^Bu′ tbbpy), 1.38 (s, 18H, ^*t*^Bu tbbpy). ^**13**^**C{**^**1**^**H} NMR (100.6 MHz, CDCl**_**3**_**):** δ 197.4 (2C, CHO), 163.9 (2C, C14′ tbbpy), 163.7 (2C, C14 tbbpy), 156.1 (2C, C12 tbbpy), 154.1 (2C, C12′ tbbpy), 152.6 (2C, C1 aryl), 151.3 (2C, CH16 tbbpy), 150.4 (2C, CH16′ tbbpy), 143.9 (2C, C2 aryl), 135.8 (2C, CH3 aryl), 124.8 (2C, CH15 tbbpy), 124.0 (2C, CH15′ tbbpy) 118.7 (2C, CH13 tbbpy), 118.2 (2C, CH13′ tbbpy), 35.7 (4C, *C*Me_3_ and C*Me*_3_′ tbbpy), 30.6 (6C, C*Me*_3_′ tbbpy), 30.4 (6C, C*Me*_3_ tbbpy). Anal. Calcd for C_44_H_52_Br_2_N_4_O_2_Pd_2_: C, 50.74; H, 5.03, N, 5.38. Found: C, 50.82; H, 4.79; N, 5.36.

### Synthesis of [C_6_H_2_{PdI(tbbpy)}_2_-1,4-(CHO)_2_-2,5] (**2b**)

Complex **I** (500 mg, 0.44 mmol), NaI (1319 mg, 8.8 mmol), and AcOH (1 mL) were added to a solution of tbbpy (472 mg, 1.76 mmol) in a 72 mL mixture of acetone and water (5:1), and the resulting suspension was refluxed for 6 h. A solid formed, which was filtered off and washed with water (3 × 10 mL) and a small amount of acetone (2 mL). The solid was then redissolved in CH_2_Cl_2_ (20 mL), stirred with MgSO_4_ for 30 min, and then filtered through additional MgSO_4_, yielding a yellow solution which was evaporated to dryness. Et_2_O (20 mL) was added to precipitate a solid, which was filtered off, thoroughly washed with Et_2_O (3 × 5 mL), and dried in vacuo to give **2b** as a yellow solid. Yield: 879 mg (89%). Mp: 217 °C (dec). IR (cm^–1^): ν(C=O): 1662. ^**1**^**H NMR (400 MHz, CDCl**_**3**_**):** δ 11.03 (s, 2H, CHO), 9.53 (d, ^3^J_HH_ = 6, 2H, H16′ tbbpy), 8.11 (s, 2H, H3 aryl), 7.99 (br s, 2H, H13′ tbbpy), 7.95 (br s, 2H, H13 tbbpy), 7.54 (dd, ^3^*J*_HH_ = 6, ^4^*J*_HH_ = 2, 2H, H15′ tbbpy) 7.38 (s, 4H, H15, 16 tbbpy), 1.45 (s, 18H, ^*t*^Bu’ tbbpy), 1.39 (s, 18H, ^*t*^Bu tbbpy). ^**13**^**C{**^**1**^**H} NMR (100.6 MHz, CDCl**_**3**_**):** δ 197.8 (2C, CHO), 163.42 and 163.37 (2C, C14,14′ tbbpy), 155.7 (2C, C12 tbbpy), 154.1 (2C, C12′ tbbpy) 152.6 (2C, CH16′ tbbpy), 150.2 (2C, CH16 tbbpy), 149.2 (2C, C1 aryl), 143.6 (2C, C2 aryl), 136.6 (2C, CH3 aryl), 124.6 (2C, CH15 tbbpy), 124.0 (2C, CH15′ tbbpy), 118.4 (2C, CH13 tbbpy), 118.1 (2C, CH13′ tbbpy), 35.54 and 35.51 (2C, *C*Me_3_ tbbpy), 30.4 (6C, C*Me*_3_′ tbbpy), 30.2 (6C, C*Me*_3_ tbbpy). Anal. Calcd for C_44_H_52_I_2_N_4_O_2_Pd_2_: C, 46.54; H, 4.62, N, 4.93. Found: C, 46.59; H, 4.64; N, 5.03.

### Synthesis of [C_6_H_2_{C(O){PdBr(tbbpy)}}_2_-1,4-(CHO)_2_-2,5] (**3a**)

CO was bubbled for 30 min through a solution of **2a** (100 mg, 0.096 mmol) in THF (20 mL) under N_2_, whereby the yellow color darkened. The mixture was then heated to 60 °C for 4 h in a CO atmosphere (whereby the color changed to red) and then filtered through MgSO_4_, yielding a red solution which was evaporated to dryness. Et_2_O (20 mL) was added to precipitate a solid, which was filtered off, thoroughly washed with Et_2_O (3 × 5 mL), and dried in vacuo to give **3a** as a pink solid. Yield: 72 mg (68%). mp 223 °C (dec). IR (cm^–1^): ν(C=O): 1682 (br).^**1**^**H NMR (400 MHz, CDCl**_**3**_**):** δ 11.01 (s, 2H, CHO, CHO″), 9.24 (d, ^3^*J*_HH_ = 6, 2H, H16′ tbbpy), 8.48 (s, 1H, H3″ aryl), 8.14 (s, 1H, H3 aryl), 7.96 (d, ^4^*J*_HH_ = 2, 2H, H13′ tbbpy), 7.95 (d, ^4^*J*_HH_ = 2, 2H, H13 tbbpy), 7.78 (d, ^3^*J*_HH_ = 6, 2H, H16 tbbpy), 7.53 (dd, ^3^*J*_HH_ = 6, ^4^*J*_HH_ = 2, 2H, H15′ tbbpy), 7.38 (dd, ^3^*J*_HH_ = 6, ^4^*J*_HH_ = 2, 2H, H15 tbbpy), 1.44 (s, 18H, ^*t*^Bu′ tbbpy), 1.38 (s, 18H, ^*t*^Bu tbbpy). ^**13**^**C{**^**1**^**H} NMR (100.6 MHz, CDCl**_**3**_**):** δ 196.3 (2C, CHO, CHO″), 168.3 (2C, C1,1″ aryl), 163.9 (2C, C14′ tbbpy), 163.7 (2C, C14 tbbpy), 155.9 (2C, C12 tbbpy), 154.1 (2C, C12′ tbbpy) 151.6 (2C, CH16 tbbpy), 150.3 (2C, CH16′ tbbpy), 144.6 (1C, CH3″ aryl), 138.7 (2C, C2,2″ aryl), 128.5 (1C, CH3 aryl), 124.7 (2C, CH15 tbbpy), 123.9 (2C, CH15′ tbbpy), 118.6 (2C, CH13 tbbpy) 118.1 (2C, CH13′ tbbpy), 35.7 (4C, *C*Me_3_ and *C*Me_3_′ tbbpy), 30.6 (6C, C*Me*_3_′ tbbpy), 30.4 (6C, C*Me*_3_ tbbpy). Anal. Calcd for C_46_H_52_Br_2_N_4_O_4_Pd_2_: C, 50.34; H, 4.78; N, 5.10. Found: C, 50.12; H, 4.52; N, 4.93.

### Synthesis of [C_6_H_2_{C(O){PdI(tbbpy)}}_2_-1,4-(CHO)_2_-2,5] (**3b**)

CO was bubbled for 30 min through a solution of **2b** (100 mg, 0.088 mmol) in THF (20 mL) under N_2_, whereby the yellow color darkened. The mixture was then heated to 60 °C for 4 h in a CO atmosphere and then filtered through MgSO_4_, yielding a pink solution, which was evaporated to dryness. Et_2_O (20 mL) was added to precipitate a solid, which was filtered off, thoroughly washed with Et_2_O (3 × 5 mL), and dried in vacuo to give **3b** as a pink solid. Yield: 76 mg (73%). mp 258 °C (dec). IR (cm^–1^): ν(C=O): 1662, 1678 cm^–1^.^**1**^**H NMR (600 MHz, CDCl**_**3**_**):** δ 10.95 (s, 2H, CHO, CHO″), 9.46 (d, ^3^*J*_HH_ = 6, 2H, H16′ tbbpy), 8.48 (s, 1H, H3″ aryl), 8.14 (s, 1H, H3 aryl), 7.96 (d, ^4^*J*_HH_ = 2, 2H, H13′ tbbpy), 7.95 (d, ^4^*J*_HH_ = 2, 2H, H13 tbbpy), 7.64 (d, ^3^*J*_HH_ = 6, 2H, H16 tbbpy), 7.50 (dd, ^3^*J*_HH_ = 6, ^4^*J*_HH_ = 2, 2H, H15′ tbbpy), 7.43 (dd, ^3^*J*_HH_ = 6, ^4^*J*_HH_ = 2, 2H, H15 tbbpy), 1.43 (s, 18H, ^*t*^Bu′ tbbpy), 1.38 (s, 18H, ^*t*^Bu tbbpy). ^**13**^**C{**^**1**^**H} NMR (150.9 MHz, CDCl**_**3**_**):** δ 196.8 (2C, CHO, CHO″), 165.8 (2C, C1,1″ aryl), 163.7 (2C, C14 tbbpy), 163.6 (2C, C14′ tbbpy), 155.8 (2C, C12 tbbpy), 154.4 (2C, C12′ tbbpy) 152.7 (2C, CH16′ tbbpy), 150.6 (2C, CH16 tbbpy), 146.5 (1C, CH3″ aryl), 138.9 (2C, C2,2″ aryl), 128.3 (1C, CH3 aryl), 124.7 (2C, CH15 tbbpy), 124.1 (2C, CH15′ tbbpy), 118.5 (2C, CH13 tbbpy) 118.2 (2C, CH13′ tbbpy), 35.8 (2C, *C*Me_3_ tbbpy), 35.7 (2C, *C*Me_3_′ tbbpy), 30.6 (6C, C*Me*_3_′ tbbpy), 30.5 (6C, C*Me*_3_ tbbpy). Anal. Calcd for C_46_H_52_I_2_N_4_O_4_Pd_2_: C, 46.37; H, 4.40, N, 4.70. Found: C, 46.69; H, 4.67; N, 4.67.

### Synthesis of 2,3,6,7-Tetrahydrobenzo[1,2-*c*:4,5-*c*′]dipyrrole-1,5-dione-2,6-dixylyl-3,7-bis{=C(NHXyl)-C(=NXyl)-[PdBr(CNXyl)_2_]} (**4**)

**3a** (300 mg, 0.29 mmol) was added to a solution of XylNC (760 mg, 5.80 mmol) in acetone under N_2_, and the resulting mixture was stirred at 50 °C for 16 h. A red solid formed, which was filtered off, washed with a small amount of acetone (2 × 3 mL), and dried in vacuo to give **4** as a red solid. Yield: 226 mg (43%). mp 217 °C. IR (cm^–1^): ν(N–H): 3376, ν(C ≡ N): 2182, ν(C=O): 1682, ν(C=N): 1614.^**1**^**H NMR (600 MHz, CDCl**_**3**_**):** δ 8.91 (s, 2H, H7), 7.18–7.08 (m, 10H, *p*-H Xyl^in3^, *p*-H Xyl^co^, *m*-H Xyl^in3^), 6.91 (d, ^3^*J*_HH_ = 8, 8H, *m*-H Xyl^co^), 6.84 (d, ^3^*J*_HH_ = 8, 4H, *m*-H Xyl^in2^), 6.83 (d, ^3^*J*_HH_ = 8, 4H, *m*-H Xyl^in1^), 6.64 (t, ^3^*J*_HH_ = 8, 2H, *p*-H Xyl^in2^), 6.60 (t, ^3^*J*_HH_ = 8, 2H, *p*-H Xyl^in1^), 5.57 (s, 2H, NH), 2.62 (s, 12H, Me Xyl^in1^), 2.49 (s, 12H, Me Xyl^in2^), 2.20 (s, 12H, Me Xyl^in3^), 2.06 (s, 24H, Me Xyl^co^). ^**13**^**C{**^**1**^**H} NMR (150.9 MHz, CDCl**_**3**_**):** δ 167.4 (2C, C=N), 165.8 (2C, C=O), 149.1 (2C, *i*-C Xyl^in1^), 142.8 (2C, C ≡ N), 138.8 (2C, C2), 138.7 (2C, *i*-C Xyl^in2^), 137.2 (2C, *i*-C Xyl^in3^), 137.0 (4C, *o*-C Xyl^in3^), 136.0 (8C, *o*-C Xyl^co^), 134.57 (4C, *o*-C Xyl^in2^), 134.59 and 130.2 (2C, C4 and C5), 130.0 (4C, *p*-CH Xyl^co^), 129.7 (2C, *p*-CH Xyl^in3^), 129.5 (4C, *o*-C Xyl^in1^), 129.29 (4C, *m*-CH Xyl^in1^), 129.26 (4C, *m*-CH Xyl^in3^), 128.9 (4C, *m*-CH Xyl^in2^), 128.0 (8C, *m*-CH Xyl^co^), 125.9 (2C, *p*-CH Xyl^in2^), 125.6 (4C, *i*-C Xyl^co^), 124.6 (2C, *p*-CH Xyl^in1^), 119.4 (2C, CH7), 112.2 (2C, C3), 21.5 (4C, Me Xyl^in1^), 20.6 (4C, Me Xyl^in2^), 19.1 (8C, Me Xyl^co^), 18.3 (4C, Me Xyl^in3^). Anal. Calcd for C_98_H_94_Br_2_N_10_O_2_Pd_2_: C, 64.80; H, 5.22; N, 7.71. Found: C, 64.53; H, 5.06, N, 7.78. Single crystals of **4**·2CH_2_Cl_2_·3hexane were grown by liquid diffusion of hexane into a solution of **4** in CH_2_Cl_2_.

### Synthesis of 2,3,6,7-Tetrahydrobenzo[1,2-*c*:4,5-*c*′]dipyrrole-1,5-dione-2,6-dixylyl-3,7-bis{=C(NHXyl)–C(O)NHXyl} (**5**)

TlOTf (98 mg, 0.28 mmol) was added to a solution of **27** (250 mg, 0.14 mmol) in commercial 1,2-dichloroethane (20 mL) under N_2_, whereby the color changed from red to black. The mixture was heated to 70 °C for 16 h, and then it was filtered through MgSO_4_, yielding a yellow solution which was concentrated in vacuo to a volume of ca. 2 mL. A small amount of Et_2_O (ca. 5 mL) was added slowly until a yellow solid started to precipitate. The mixture was left in an ice bath for 24 h, and then it was filtered through Celite, again yielding a yellow solution, which was evaporated in vacuo to dryness. Hexane (15 mL) was added to precipitate a solid, which was filtered off, washed with hexane (3 × 5 mL) and a small amount of cold Et_2_O (1 mL), and dried in vacuo to give **5** as a yellow solid. Yield: 25 mg (56%). mp 217 °C. IR (cm^–1^): ν(N–H): 3369, ν(C=O): 1674 (br). ^**1**^**H NMR (400 MHz, CDCl**_**3**_**):** δ 8.34 (s, 2H, H7), 7.25–7.17 (m, 6H, *m*,*p*-H Xyl^3^), 7.06–7.00 (m, 2H, *p*-H Xyl^1^), 7.00–6.92 (m, 10H, *m*-H Xyl^1^, *m*,*p*-H Xyl^2^), 6.89 (s, 2H, NH^1^), 5.12 (s, 2H, NH^2^), 2.22 (s, 12H, Me Xyl^3^), 2.21 (s, 12H, Me Xyl^2^), 1.70 (s, 12H, Me Xyl^1^). ^**13**^**C{**^**1**^**H} NMR (100.6 MHz, CDCl**_**3**_**):** δ 164.8 (2C, CO^6^), 161.7 (2C, CO^1^), 137.6 (4C, *o*-C Xyl^3^), 137.2 (2C, *i*-C Xyl^2^), 135.8 (2C, *i*-C Xyl^3^), 135.6 (4C, *o*-C Xyl^2^), 135.2 (4C, *o*-C Xyl^1^), 133.4 (2C, C4 or C5), 132.5 (2C, *i*-C Xyl^I^), 130.7 (2C, C5 or C4), 130.0 (2C, *p*-CH Xyl^3^), 129.3 (4C, *m*-CH Xyl^2^), 129.2 (4C, *m*-CH Xyl^3^), 128.7 (4C, *m*-CH Xyl^1^), 127.7 (2C, *p*-CH Xyl^1^), 127.4 (2C, C2), 126.9 (2C, *p*-CH Xyl^2^), 118.2 (2C, CH7), 113.4 (2C, C3), 18.9 (4C, Me Xyl^2^), 18.4 (4C, Me Xyl^3^), 18.1 (4C, Me Xyl^2^). Exact mass (HR ESI + TOF): calcd for **5** + H^+^ (C_62_H_61_N_6_O_4_) *m*/*z* 953.4749; found, 953.4758, Δ = 0.99 ppm. Single crystals of **5**·2CDCl_3_·were grown by the slow evaporation of a solution of **5** in CDCl_3_.

## Supporting Information Available

Details of the synthesis and characterization of complex I, X-ray structure determination data, and NMR spectra (PDF)

## Footnotes

a Indeed, the APT spectrum of **2b**, when processed without window function, shows a significant broadening of the 13C resonances of the CHO/CHO″, C1/C1″, and C2/C2″ carbons but not of those of the tbbpy ligand.
