# Supplementary material for: Synthesis and Reactivity of Dipalladated Derivatives of Terephthalaldehyde
Source: Organometallics. 2024 Jul 24;43(15):1647–57. doi: 10.1021/acs.organomet.4c00231 (PMC11323953; doi:10.1021/acs.organomet.4c00231)
Supplement: Supplementary file 1 — om4c00231_si_001.pdf [file om4c00231_si_001.pdf]

Supporting Information-

Synthesis and Reactivity of Dipalladated Derivatives of Terephthalaldehyde

María-José Fernández-Rodríguez,† Peter G. Jones,‡ José Vicente,†,* Eloísa Martínez-Viviente†,*

† Grupo de Química Organometálica, Departamento de Química Inorgánica, Facultad de Química, Universidad de Murcia, E-30071 Murcia, Spain. E-mails: jvs1@um.es; eloisamv@um.es

‡ Institut für Anorganische und Analytische Chemie, Technische Universität Braunschweig, Hagenring 30, 38106 Braunschweig, Germany. E-mail: p.jones@tu-braunschweig.de

● Synthesis and characterization of [{μ-*C1,C4,N,N”*-C_6H_2{C(H)=N(^{n}Bu)}$_2$-2,5}{Pd(μ-OAc)}]$_2$ (**I**) S2

● X-ray structure determinations S3

● ^{1}H and APT NMR spectra for **1**–**5**. Individual reaction schemes. S6

Synthesis and characterization of [{μ-*C1,C4,N,N''*-C_6H_2{C(H)=N(^{n}Bu)}$_2$-2,5}{Pd(μ-OAc)}]$_2$ (I)[1]

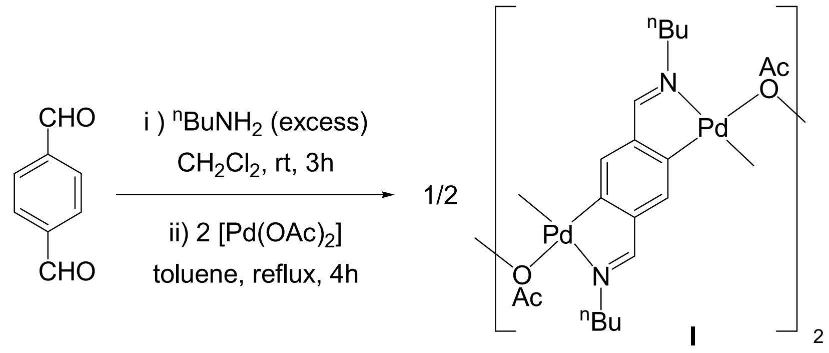

A solution of ^{n}BuNH$_2$ (1.00 g, 7.45 mmol) and terephthalaldehyde (250 mg, 1.86 mmol) in CH_2Cl_2 (3 mL) was stirred for 1.5 h. The solvent and the excess amine were then evaporated *in vacuo*, leaving a yellow oil to which [Pd(OAc)]$_2$ (877 mg, 3.91 mmol) and toluene (60 mL) were added. The mixture was refluxed for 4 h in a CaH_2-containing Soxhlet, and then it was concentrated *in vacuo*. The residue was extracted with CH_2Cl_2 (60 mL) and the extract was filtered over Celite. The resulting red solution was concentrated *in vacuo* to ca. 3 mL. Et_2O (25 mL) was added forming an orange suspension which was filtered off, washed with Et_2O (3x10 mL), and dried *in vacuo* to give **I** as an orange solid, which is soluble in CH_2Cl_2 and acetone. For a complete purification the solid was dried in an oven at 70ºC for 24 h and then in a desiccator over P_2O_5 for 5 days. Yield: 826 mg (77%). Mp: 240 ºC (dec). IR (cm^{-1}): ν(C=O): 1576; ν(C=N): 1556. ^{1}H NMR (300 MHz, $CDCl_3$): 7.62 (s, 2H, HC=N), 6.48 (s, 2H, aryl), 3.75-3.55 and 3.25-3.05 (m, 2H, CH_2 nBu), 2.19 and 2.00 (s, 3H, $MeCO_2$), 1.95-1.65 (m, 4H, CH_2 nBu), 1.50-1.15 (m, 4H, CH_2 nBu), 0.93 (t, $^3J_{HH}$ = 7 Hz, 6H, Me nBu). ^{13}C{^{1}H} NMR (75.4 MHz, $CDCl_3$): 181.2 and 179.7 (Me*C*O$_2$), 172.6 (C=N), 152.0 and 145.3 (aryl C), 129.9 (aryl CH), 59.7, 31.7, and 19.8 (CH_2 nBu), 24.5 and 24.3 (*Me*CO$_2$), 13.6 (Me nBu). Anal. Calcd for $C_{20}H_{28}N_2O_4Pd_2$: C, 41.90; H, 4.93; N, 4.89. Found: C, 42.17; H, 5.00, N, 4.88.

[1] F.-S. Hernández, PhD Thesis, University of Murcia, 2001.

X-Ray Structure Determinations

Crystals were mounted in inert oil on glass fibres. Intensity data were recorded on a Bruker SMART 1000 CCD (**1**·4$CHCl_3$, **4**·2CH_2Cl_2·3hexane), or a Bruker APEX-2 diffractometer (**5**·2$CDCl_3$) using monochromated Mo *K*α radiation. Absorption corrections were based on multi-scans (but were not performed for **5**·2$CDCl_3$ because they led to no improvement). Hydrogen atoms of NH groups were refined freely but with distance restraints. Other hydrogen atoms were included using rigid methyl groups or a riding model. Structures were refined anisotropically on F^2 using the program SHELXL-2019 (G. M. Sheldrick, University of Göttingen, Germany). *Special features and exceptions*: In structure **1**·4$CHCl_3$, both chloroform molecules, the triflate anion and one *t*-butyl group are disordered. The dataset for **4**·2CH_2Cl_2·3hexane was of limited resolution because of the large amount of solvent; the hexane molecules in particular displayed high *U* values. For **5**·2$CDCl_3$ the $CDCl_3$ molecule was disordered over two sites with relative occupations 0.825, 0.175(3). The disorder models were refined with appropriate restraints to improve refinement stability, but the dimensions of disordered groups should always be interpreted with caution.

Crystallographic data are summarized in Table S1. Additionally, complete data have been deposited with the Cambridge Crystallographic Data Centre under the numbers CCDC-2345291 (**1**·4$CHCl_3$), -2345292 (**4**·2CH_2Cl_2·3hexane) and -2345293 (**5**·2$CDCl_3$). Copies of the data can be obtained free of charge from www.ccdc.cam.ac.uk/data_request/cif.

Table S.1. Crystal data and structure refinements for compounds **1**·$4CHCl_3$, **4**·$2CH_2Cl_2$·3hexane, and **5**·$2CDCl_3$

	**1**·$4CHCl_3$	**4**·$2CH_2Cl_2$·3hexane	**5**·$2CDCl_3$
Empirical Formula	$C_{58}H_{74}Cl_{12}F_6N_6O_6Pd_2S_2$	$C_{118}H_{140}Br_2Cl_4N_{10}O_2Pd_2$	$C_{64}H_{60}D_2Cl_6N_6O_4$
Formula Weight	1767.55	2244.82	1193.91
Temperature	133(2) K	133(2) K	100(2) K
Wavelength	0.71073 Å	0.71073 Å	0.71073 Å
Crystal system	Triclinic	Monoclinic	Triclinic
Space group	P(-1)	$P2_1/n$	P(-1)
Cell constants			
a (Å)	11.9565(7)	16.1438(19)	8.7235(8)
b(Å)	12.5085(8)	19.885(2)	12.5281(12)
c (Å)	13.4126(8)	18.120(2)	14.1188(14)
α (deg)	105.376(3)	90	101.419(4)
β (deg)	94.960(3)	103.313(3)	94.655(4)
γ (deg)	102.884(3)	90	99.974(4)
Volume ($Å^3$), *Z*	1862.7(2), 1	5660.5(11), 2	1478.9(2), 1
Density (calculated)	1.576 Mg m^{-3}	1.317 Mg m^{-3}	1.340 Mg m^{-3}
Absorption coefficient	1.0 mm^{-1}	1.2 mm^{-1}	0.34 mm^{-1}
F(000)	894	2328	622
Crystal size (mm)	0.40 x 0.30 x 0.15	0.40 x 0.30 x 0.15	0.25 x 0.15 x 0.13
θ range (deg)	1.59 - 30.03	1.52 - 25.53	2.39 - 30.51
Index ranges	$-16 \le h \le 16$<br>$-17 \le k \le 17$<br>$-18 \le l \le 18$	$-19 \le h \le 19$<br>$-24 \le k \le 24$<br>$-21 \le l \le 21$	$-12 \le h \le 12$<br>$-17 \le k \le 17$<br>$-20 \le l \le 20$
Reflections collected	37843	47230	37642
Independent reflections	10792	10445	9012
R_{int}	0.0329	0.118	0.0268
abs corr	Semi-empirical from equivalents	Semi-empirical from equivalents	None
Transmissions	0.860 - 0.742	0.844 - 0.590	
Refinement method	Full-matrix least-squares on F^2	Full-matrix least-squares on F^2	Full-matrix least-squares on F^2
Data/restraints/params	10792 / 400 / 547	10445 / 912 / 621	9012 / 40 / 388
Goodness-of-fit on F^2	1.23	1.05	1.03
Final *R* indices (I>2σ(I))			
*R*1	0.0685	0.0805	0.0527
*wR*2	0.1840	0.1794	0.1345
R indices (all data)			
*R*1	0.0806	0.1410	0.0686
*wR*2	0.1889	0.2046	0.1463
Largest diff peak (e $Å^{-3}$)	1.45	1.34	0.88
Largest diff hole (e $Å^{-3}$)	-1.42	-1.17	-0.74

^{1}H and APT NMR spectra for the complexes 1-4. Individual reaction schemes.

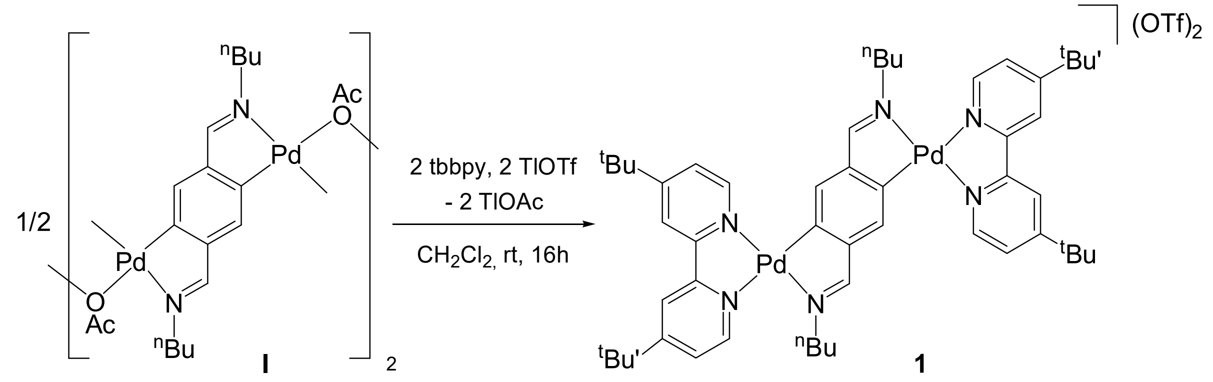

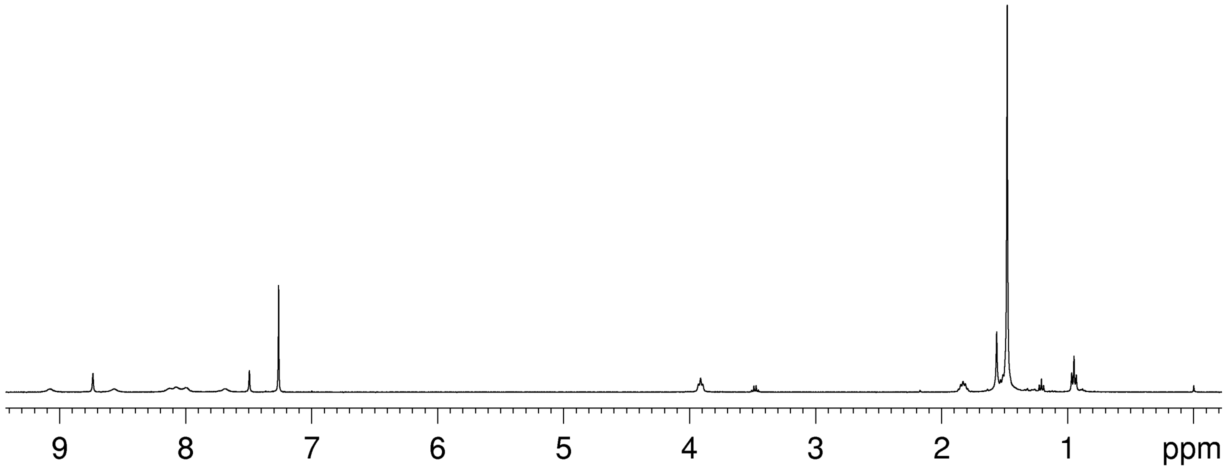

Figure S1. ^{1}H-NMR spectrum (400 MHz, $CDCl_3$) of compound **1**

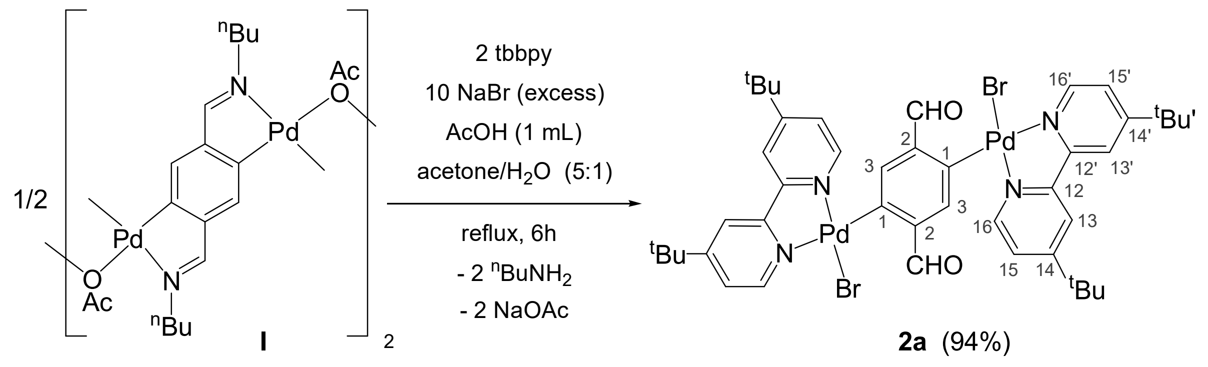

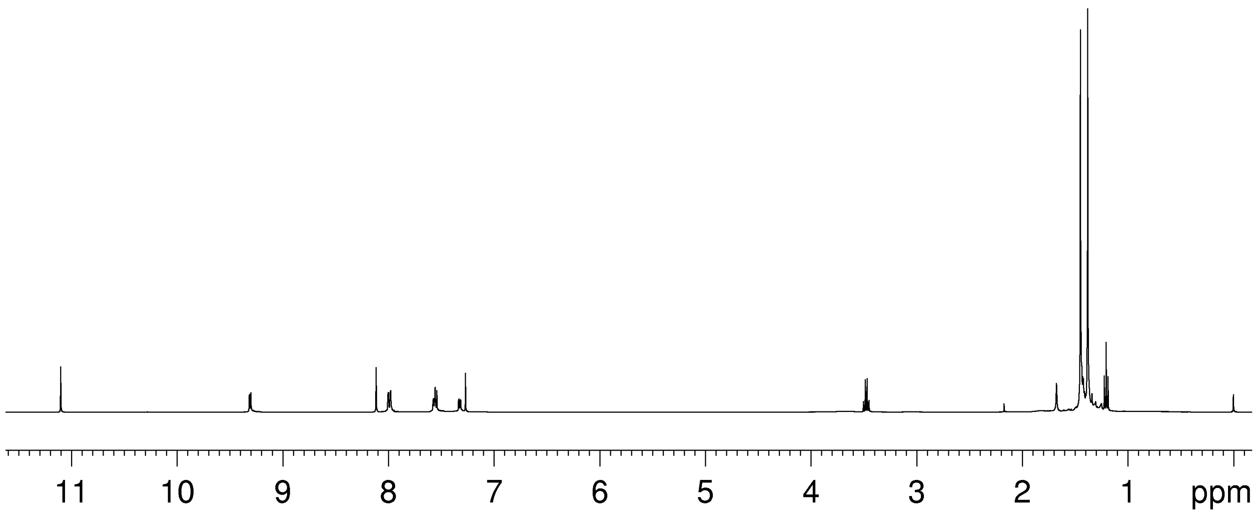

Figure S2. ^{1}H-NMR spectrum (400 MHz, $CDCl_3$) of compound **2a**

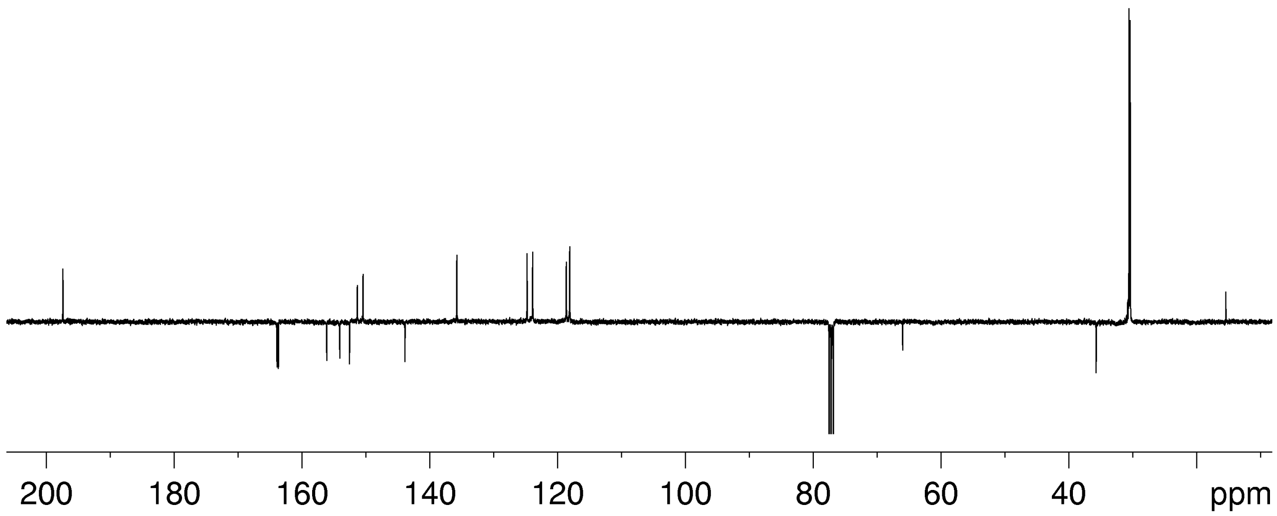

Figure S3. APT spectrum (100.6 MHz, $CDCl_3$) of compound **2a**

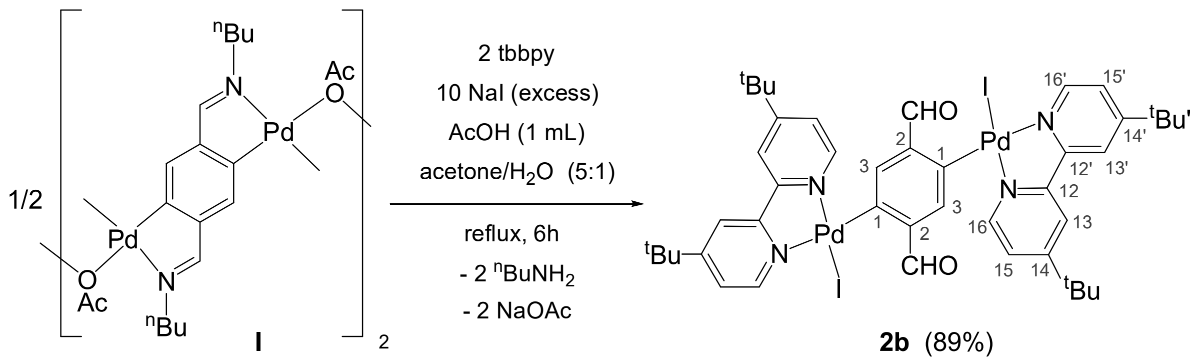

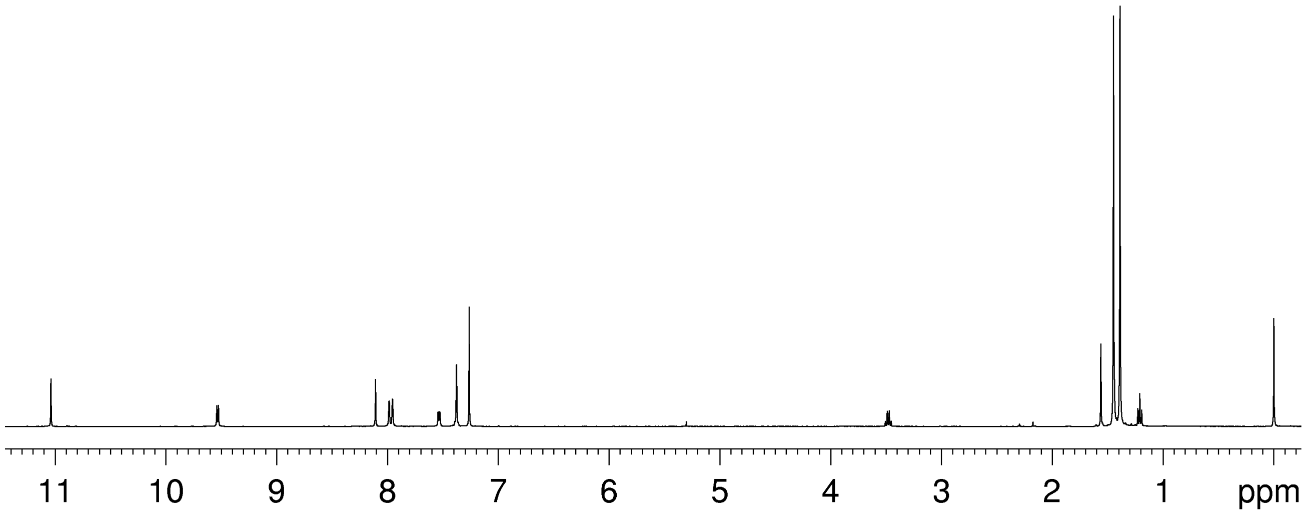

Figure S4. ^{1}H-NMR spectrum (400 MHz, $CDCl_3$) of compound **2b**

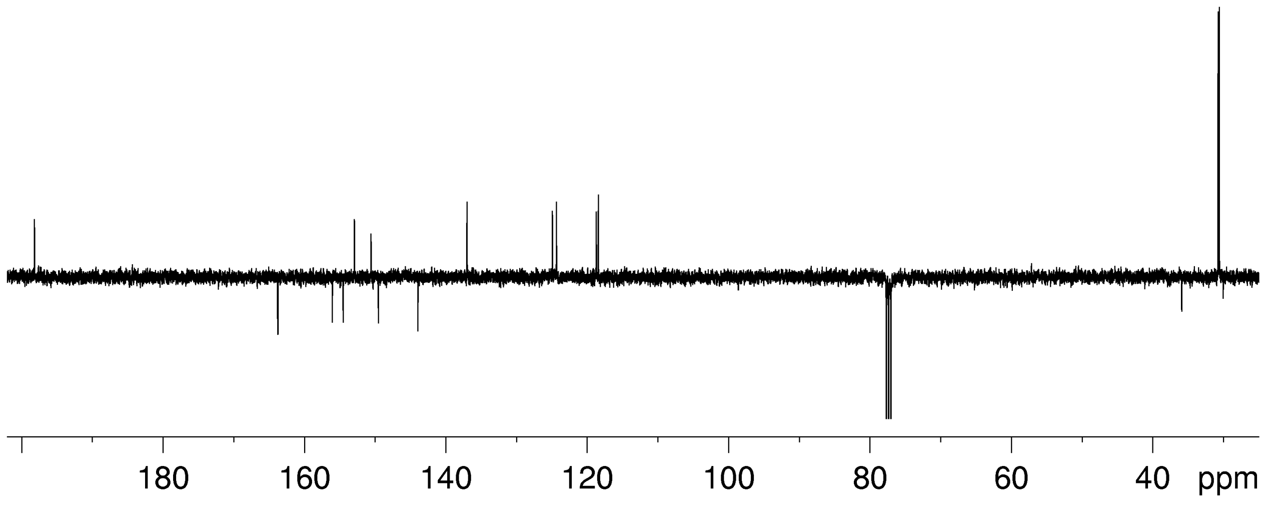

Figure S5. APT spectrum (100.6 MHz, $CDCl_3$) of compound **2b**

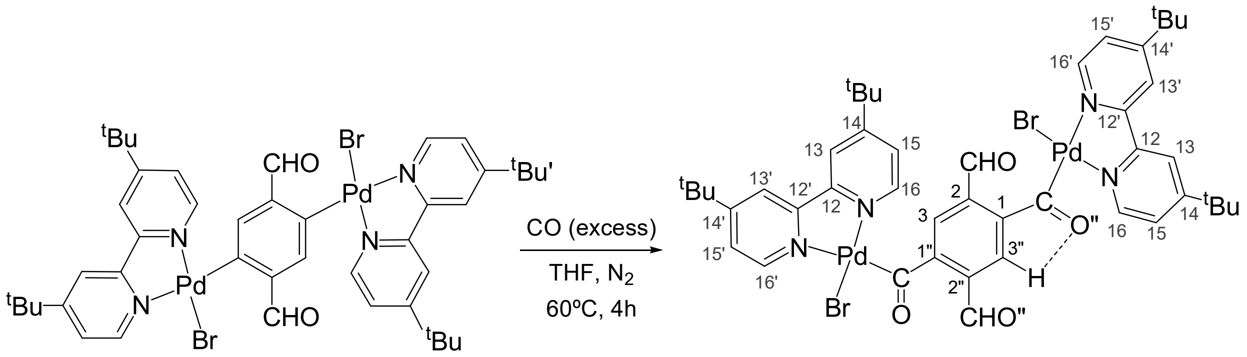

2a **3a** (68%)

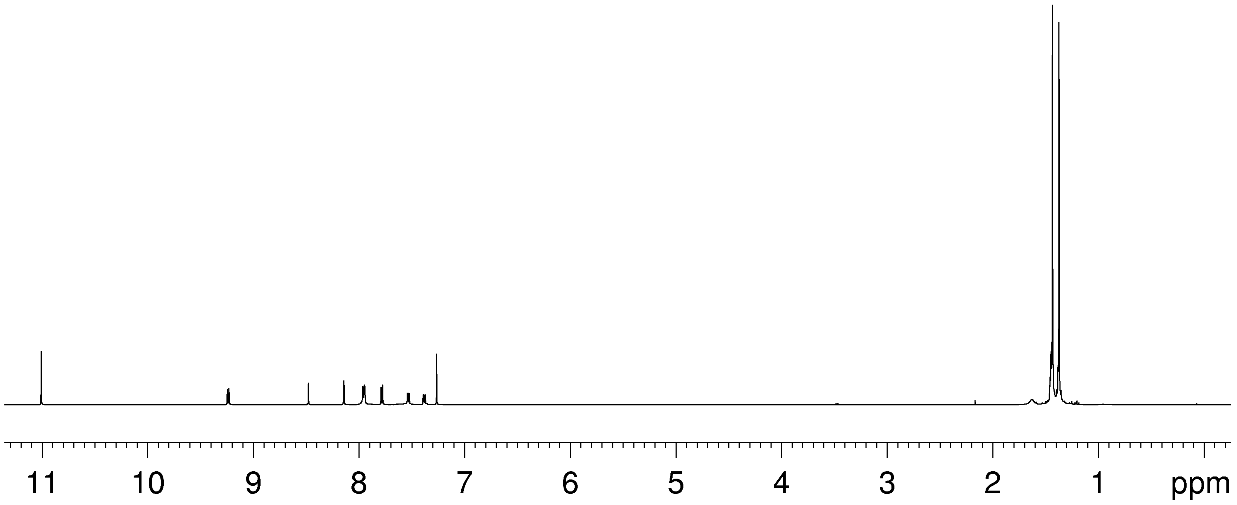

Figure S6. ^{1}H-NMR spectrum (400 MHz, $CDCl_3$) of compound **3a**

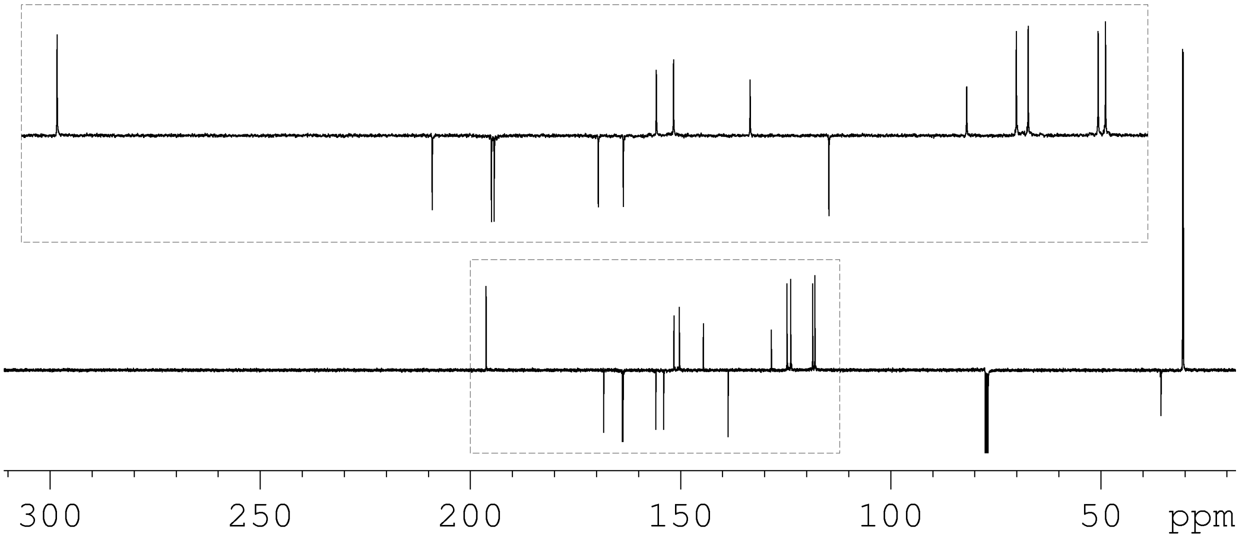

Figure S7. APT spectrum (100.6 MHz, $CDCl_3$) of compound **3a**

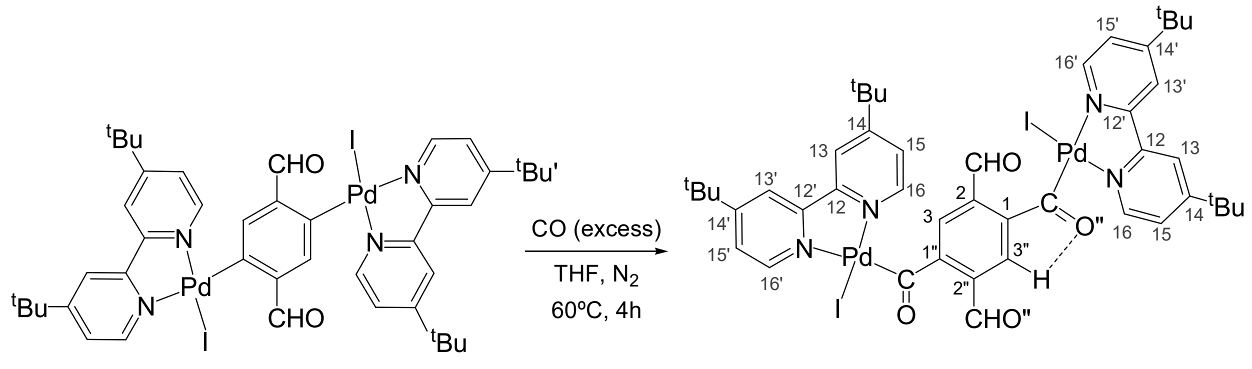

2b **3b** (73%)

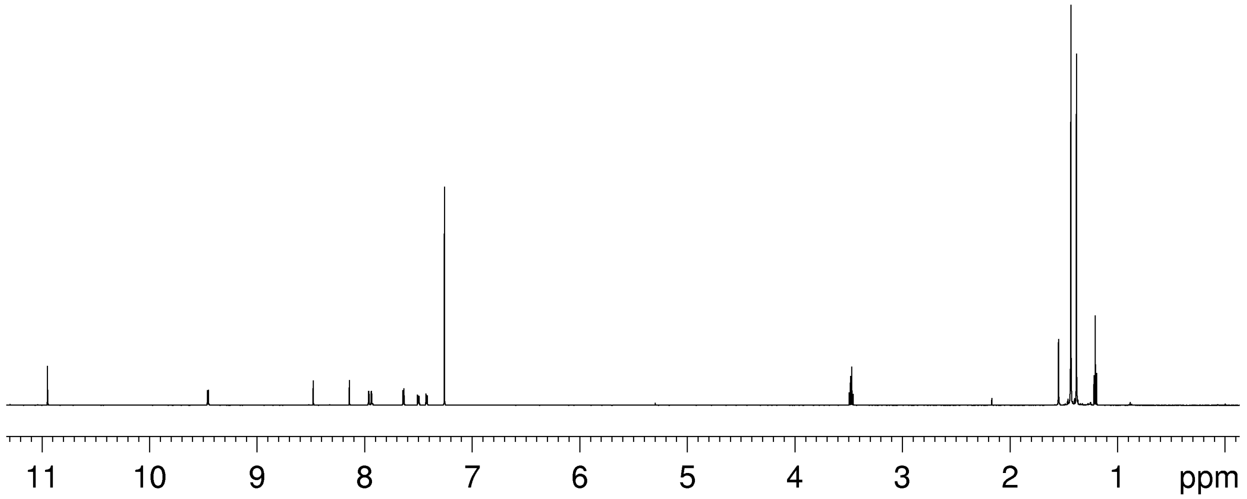

Figure S8. ^{1}H-NMR spectrum (600 MHz, $CDCl_3$) of compound **3b**

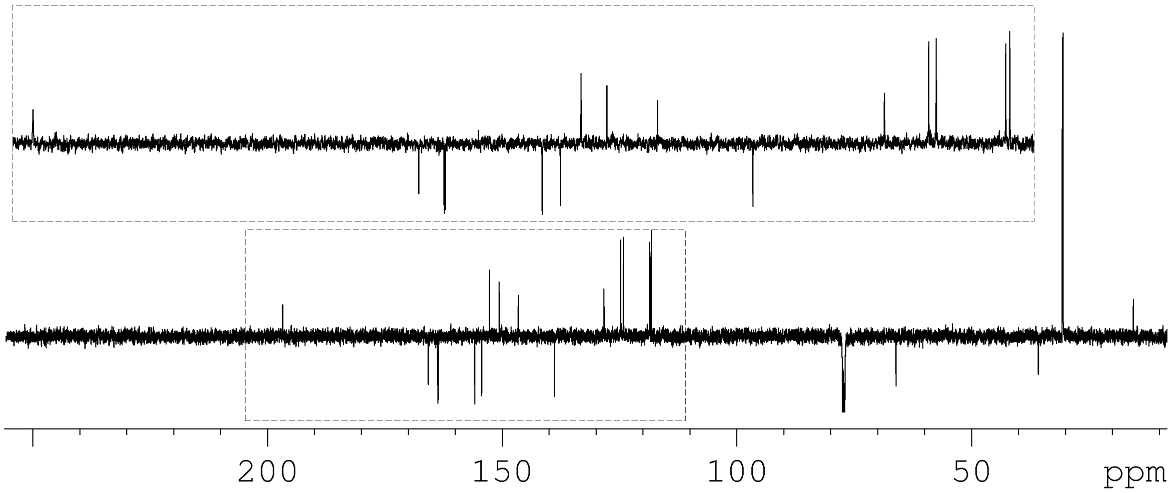

Figure S9. APT spectrum (150.9 MHz, $CDCl_3$) of compound **3b**

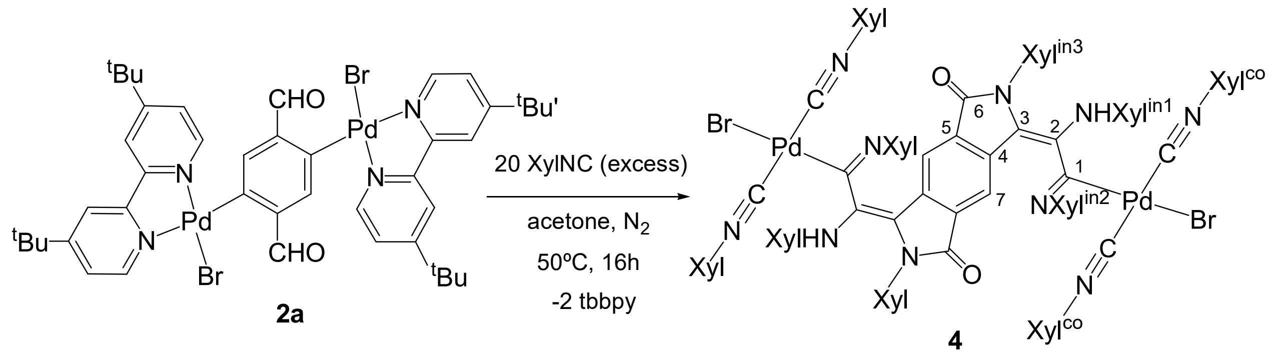

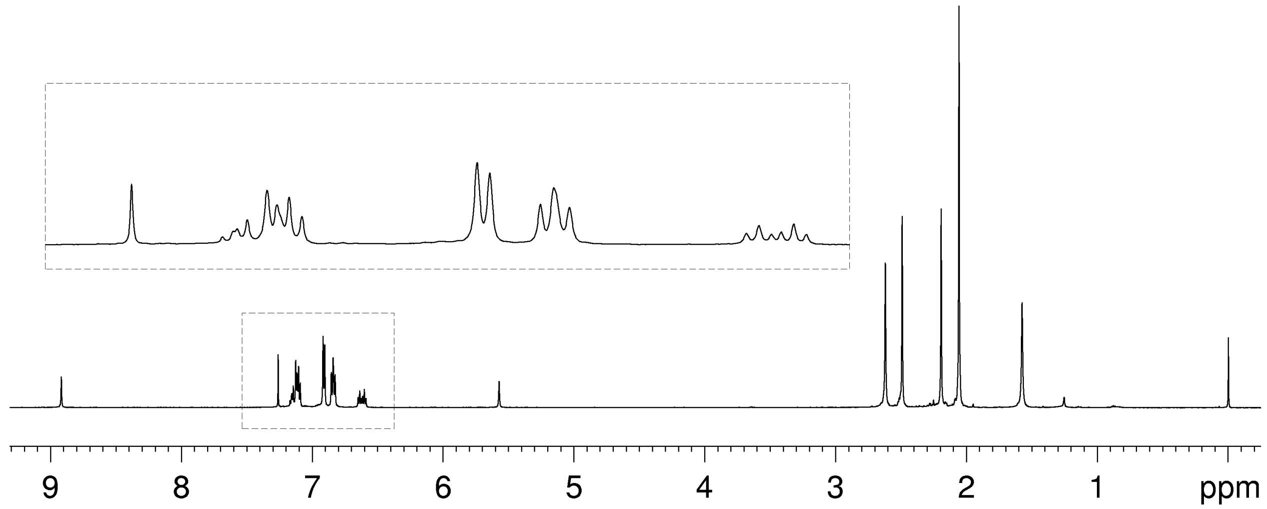

Figure S10. ^{1}H-NMR spectrum (600 MHz, CDCl$_3$) of compound **4**

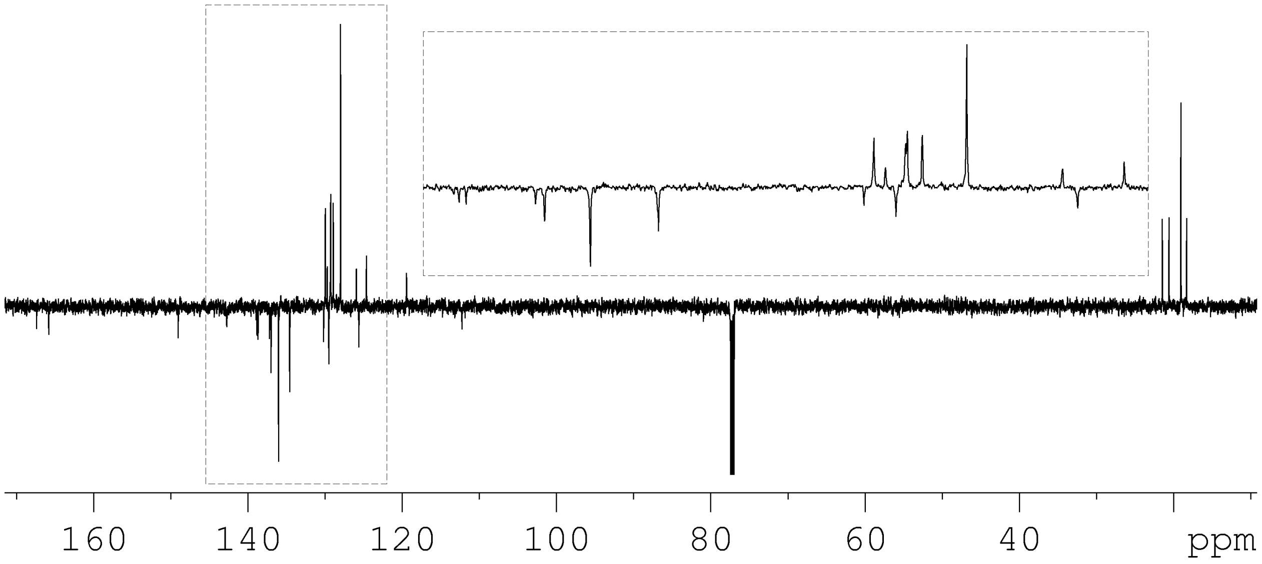

Figure S11. APT spectrum (150.9 MHz, CDCl$_3$) of compound **4**

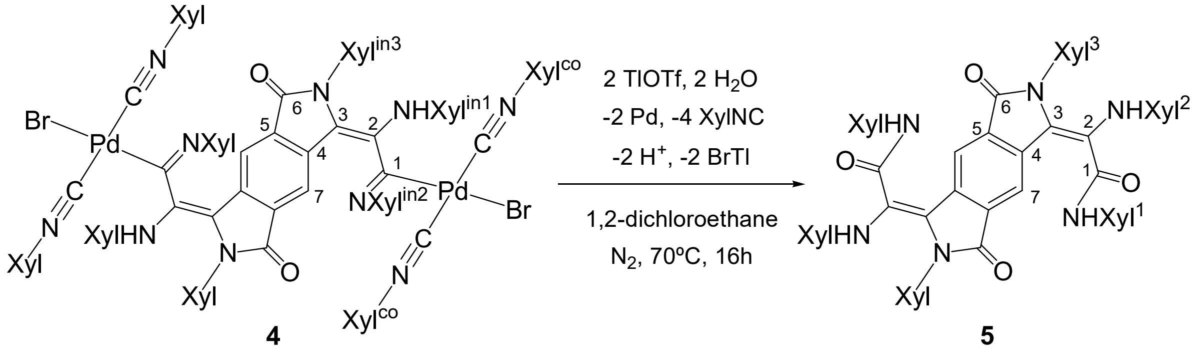

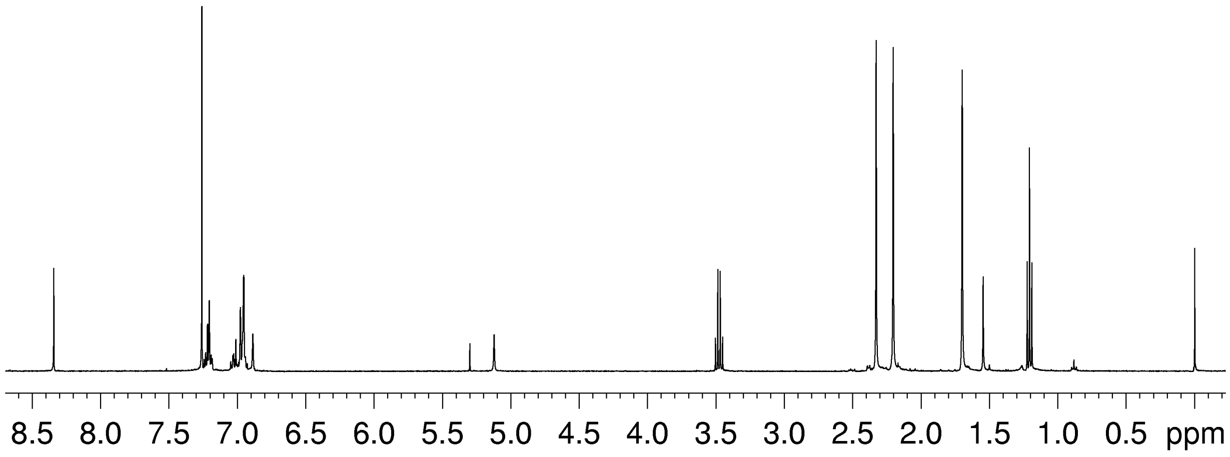

Figure S12. ^{1}H-NMR spectrum (600 MHz, $CDCl_3$) of compound **5**

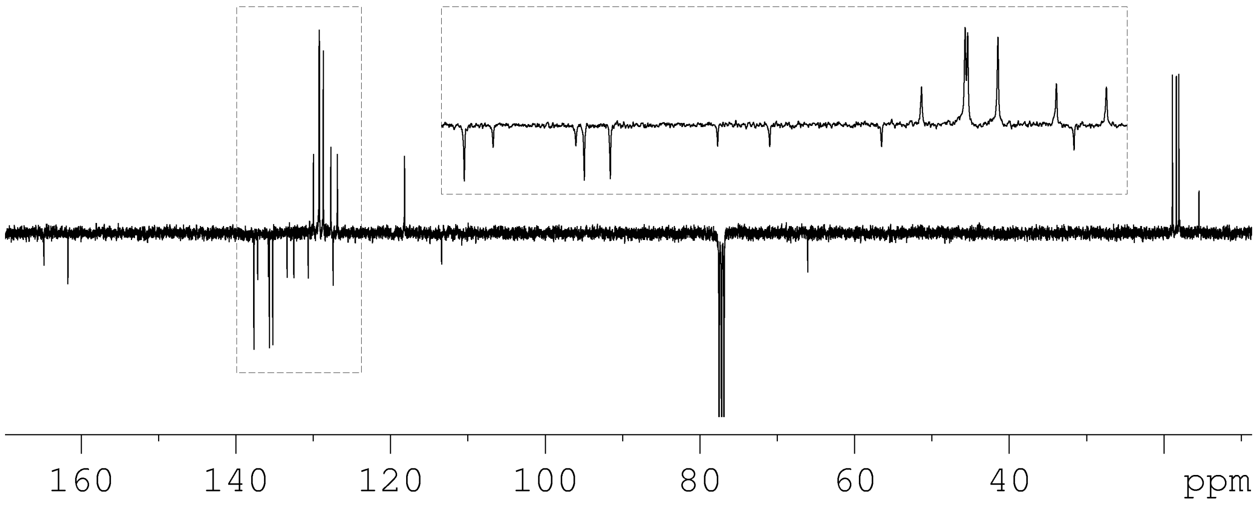

Figure S13. APT spectrum (150.9 MHz, $CDCl_3$) of compound **5**
